# Spatial domains identification in spatial transcriptomics using modality-aware and subspace-enhanced graph contrastive learning

**DOI:** 10.1016/j.csbj.2024.10.029

**Published:** 2024-10-22

**Authors:** Yang Gui, Chao Li, Yan Xu

**Affiliations:** a School of Mathematics and Physics, University of Science and Technology Beijing, Beijing, 100083, China; b School of Statistics and Applied Mathematics, Anhui University of Finance and Economics, Bengbu, 233041, China

**Keywords:** Spatial transcriptomics, Spatial domain identification, Graph contrastive learning, Subspace analysis

## Abstract

Spatial transcriptomics (ST) technologies have emerged as an effective tool to identify the spatial architecture of tissues, facilitating a comprehensive understanding of organ function and the tissue microenvironment. Spatial domain identification is the first and most critical step in ST data analysis, which requires thoughtful utilization of tissue microenvironment and morphological priors. Here, we propose a graph contrastive learning framework, GRAS4T, which combines contrastive learning and a subspace analysis model to accurately distinguish different spatial domains by capturing the tissue microenvironment through self-expressiveness of spots within the same domain. To uncover the pertinent features for spatial domain identification, GRAS4T employs a graph augmentation based on histological image priors, preserving structural information crucial for the clustering task. Experimental results on eight ST datasets from five different platforms show that GRAS4T outperforms five state-of-the-art competing methods. Significantly, GRAS4T excels at separating distinct tissue structures and unveiling more detailed spatial domains. GRAS4T combines the advantages of subspace analysis and graph representation learning with extensibility, making it an ideal framework for ST domain identification.

## Graphical abstract

**Figure:**
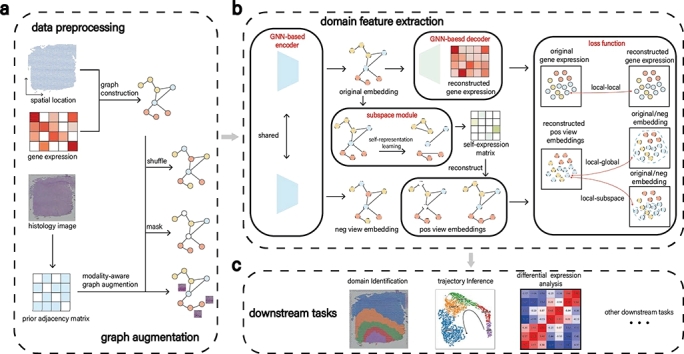

## 1 Introduction

The emergence of spatial genomics has revolutionized biological and medical research, facilitating an unprecedented understanding of functional arrangement. Knowledge of the relative locations of different cells in a tissue is critical for understanding tissue development because spatial information helps to link gene-coded tissue structural development to different functional domains. Deciphering spatial domains, i.e., identifying spatial spots with coherent gene expression and histology, is considered a critical step in spatial transcriptomics analyses. It aims to assign spatial data into a series of meaningful clusters, where each cluster is considered a spatial domain [1]. The successful identification of spatial domains usually relies on the effective utilization of gene expression data, spatial location, and the corresponding tissue image [2], [3], offering the potential to unveil inherent interactions and characterize the tissue microenvironment.

Several computational tools have been developed to decipher spatial domains. These methods can be classified as non-spatial- and spatial-based methods, depending on whether or not they incorporate the spatial information. Among the non-spatial-based methods, classical clustering methods such as Louvain [4], mclust [5], and kmeans++ [6] have been proposed. These methods neglect spatial information and histology, leading to inconsistent identification of domains and reduced accuracy compared to spatial-based methods like BayesSpace [7], Giotto [8], and SC-MEB [9], etc. In addition to the machine learning methods mentioned earlier, several deep learning-based methods [10], [11], [12], [13], [14], [15] have also been developed within the categories of spatial-based methods. Notably, there have been extensive research on methodologies based on Graph Neural Network (GNN). Since spatial transcriptomics data inherently include spatial location that naturally link individual spots, the task of spatial domain identification can be transformed into a node classification problem, which GNNs have been proposed to solve. GNN-based methods convert the ST data into graph structures, enhancing data quality for downstream tasks by extracting deep graph structure information. Many GNN-based models employ an autoencoder architecture or a contrastive strategy to learn the representation of each spot. Particularly, the graph attention autoencoder framework STAGATE [14] employs a cell type-aware attention mechanism to learn low-dimensional embeddings by integrating spatial information and gene expression profiles. DeepST [11] identifies spatial domains based on a denoising autoencoder and a graph autoencoder. By integrating spatial local relations between gene expression, spatial location, and histology images, it enhances gene expression data, which is further fed into a deep representation learning model to learn low-dimensional representation. The graph contrastive learning-based methods provide new perspectives on spatial transcriptomics data analysis. CCST, as proposed by Li et al. [12], employs gene expression profiles and hybrid adjacency matrices based on cellular neighborhoods as inputs to a neural network that encodes cellular embeddings from spatial transcriptomics data. SpaceFlow [13] ensures the distance between learned potential embeddings mimics the distance between the gene expressions of a spot or cell, as well as the distance between spatial locations. conST [15] uses three types of spatial transcriptomics data and takes into account the global, local, and contextual information of the graph. By leveraging this information, conST effectively learns the underlying graph structure of the data. While these methods account for nearest neighbor relationships between spots, they fall short in adaptively distinguishing or identifying truly related spots. As a result, they struggle to precisely demarcate spatial domains. Moreover, most of these methods use domain-agnostic graph augmentations (DAGA) that do not consider spatial segmentation-related information, leading to poor discriminative representation [16], [17].

To address the challenges mentioned above, we propose GRAS4T, a modality-aware GRAph contraStive learning based on Subspace-enhanced for Spatial domain identification in Spatial Transcriptomic. GRAS4T initially incorporates a morphology prior-based graph augmentation module, which guides the formation and removal of edges within the spot-spot graph. The module constructs positive views that retain morphological information, enabling the GNN to learn domain segmentation-related representation by way of contrast. For adaptively obtaining real spot nearest neighbor information, GRAS4T incorporates the subspace module into the contrastive learning framework. Unlike the subspace model alone, GRAS4T uses a latent feature space to obtain a self-expression matrix with better block diagonal properties [18]. With the self-expression matrix, the contrastive view contains a realistic tissue microenvironment adaptively, thus obtaining more features relevant to the downstream clustering task. It is noteworthy that the subspace module can be deployed as a plug-and-play module within existing graph contrastive learning frameworks. Utilizing this module in more advanced graph contrastive learning models can further enhance the effectiveness of the algorithms. GRAS4T is applicable to various platforms (e.g., 10x Visium, Stereo-seq, MERFISH, etc.) and different tissues (e.g., brain, breast, olfactory bulb, etc.), and is extensible due to its combination of traditional machine learning models and advanced deep learning techniques utilizing graph neural networks.

## 2 Materials and methods

### 2.1 Overall architecture of GRAS4T

**Fig. 1:**
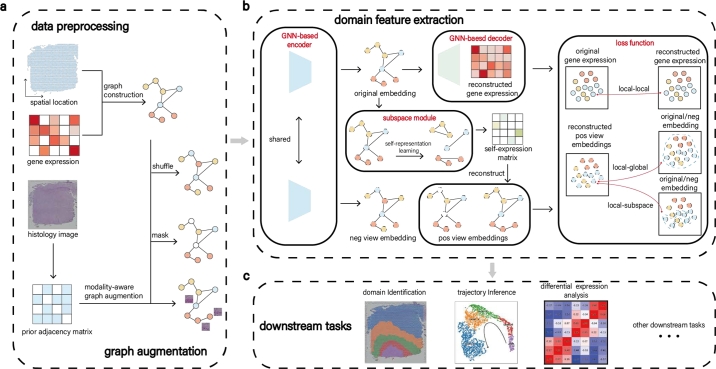
Overview of GRAS4T. (a) GRAS4T constructs the graph G from gene expression profiles and spatial locations, then applies masking and shuffling to augment it, generating positive and negative views. The prior adjacency matrix is derived from the hematoxylin and eosin (H&E) stained images, while the positive view is obtained by the augmenting graph G with the help of the prior adjacency matrix. The graph G along with the three generated views will serve as inputs to the GNN. (b) GRAS4T designs a graph contrastive learning framework, employing a GNN-based encoder, a GNN-based decoder, and a subspace module. This method effectively extracts potential representation via contrastive learning loss and reconstruction loss. (c) The potential embeddings learned through GRAS4T are essential for domain identification, trajectory inference, and differential expression analysis.

GRAS4T uses the graph contrastive learning framework as its backbone, incorporating modality-aware graph augmentation and a subspace module to distinguish tissue boundaries. As illustrated in Fig. 1, the architecture of our GRAS4T contains four components, namely **data preprocessing, graph augmentation, domain feature extraction, and downstream task**. Given an input graph G (defined in Supplementary Section 1.1), it employs graph augmentation techniques to obtain the positive pairs and uses the corruption function to obtain the negative pairs. These graphs serve as inputs to the GNN-based encoder to obtain low-dimensional representations (Fig. 1a). Next, the node representation obtained from the input graph G is fed into the subspace module to generate the self-expression matrix. Unlike the classic contrastive learning structure, GRAS4T adopts the self-expression matrix to obtain the reconstructed representation of positive views, which participates in the computation of the local-subspace contrastive loss. Furthermore, GRAS4T uses the decoder to recover the data matrix so that the embedding retains sufficient gene expression-related information. The entire model is trained using local-global contrastive loss, local-subspace contrastive loss, and reconstruction loss (Fig. 1b). Finally, the node representation is applied for downstream tasks such as clustering (Fig. 1c). Detailed explanations of these modules will be elaborated in the subsequent sections.

### 2.2 Data preprocessing

The spatial transcriptomics data are preprocessed before converting them to graph structure data for the GRAS4T (Fig. 1a). Specifically, GRAS4T takes gene expression, spatial location, and histology image (when available) as input. The top 3,000 variable genes are selected based on the raw gene expression profiles [14]. It should be noted that in the STARmap dataset, with less than 3,000 genes, no gene filtering is applied. For the MERFISH dataset, blank genes and Fos genes (non-numerical values) are removed. Additionally, cells labeled with ‘Ambiguous’ are also filtered out by following the standard protocol proposed in Abdelaal et al. [19]. The selected gene expressions of top genes are then normalized based on the library size and subsequently log-transformed. We denote the preprocessed gene expression data as $X\in R^{d\times n}$, where *d* is the number of genes and *n* is the number of spots or cells. In constructing the adjacency matrix, spatial location can be effective for identifying contiguous domains. However, for discontinuous domains, spatial location alone is insufficient to construct edge relationships. To address this issue, the spatial location information and the gene expression are used to construct the adjacency matrix $A\in R^{n\times n}$ where $A_{ij}\neq 0$ if a linkage exists between spot *i* and spot *j*, otherwise $A_{ij}=0$. Particularly, GRAS4T selects $k_{S}$ and $k_{E}$ nearest neighbors to construct adjacency matrices $A_{S},A_{E}$, which are based on spatial information and gene expression, respectively. These two matrices are then merged using a balancing hyperparameter *α*, expressed as $A=(1-\alpha )A_{S}+\alpha A_{E}$. Accordingly, the input graph G can be described by the gene expression matrix **X** and the adjacency matrix **A**.

### 2.3 Modality-aware graph augmentation module

Graph augmentation involves attribute masking and edge perturbation [20], both of which are random augmentations on a global scale (Supplementary Section 1.1). Inspired by [16], [21], a modality-aware graph augmentation strategy is designed. Specifically, GRAS4T utilizes H&E image information to randomly perturb the edges of the input graph G, thereby preserving information relevant to domain segmentation (Fig. 1a).

To obtain the morphology feature of the tissue, GRAS4T first tiles the H&E image into the patches. Specifically, GRAS4T centers each patch on the spot, cropping it into a 50×50 pixel patch, which is subsequently uniformly resized to 224×224 pixels. GRAS4T then uses a pretrained Convolutional Neural Network to extract the latent representation $X_{I}$ from the patches. GRAS4T employs *k* nearest neighbor to construct the nearest neighbor graph $A_{I}$, which then guides the augmentation of the nearest neighbor graph **A**. The graph augmentation function τ(⋅) is applied to graph G to obtain the augmentation view. Specifically, the positive augmentations for nodes and edges can be represented as $\tau_{X}(\cdot )$ and $\tau_{A}(\cdot )$, respectively. Here, the morphology prior-based edge perturbation $\tau_{A}(A)$ is defined as

(1) $$\tau_{A}(A)=A⊙(1-L⊙(A-A⊙A_{I}))+(1-A)⊙(L⊙(A_{I}-A⊙A_{I})),$$

where ⊙ is the Hadamard product (element-wise product). $L\in R^{n\times n}$ is a random perturbation location matrix, $L_{ij}=L_{ji}=1$, if the connection between spot *i* and spot *j* is perturbed, otherwise $L_{ij}=L_{ji}=0$. The two terms in $\tau_{A}(A)$ represent edge drop and addition in **A**, respectively. The first term represents a randomly disconnected link between spots with dissimilar tissue structures but similar spatial locations or gene expression, and the second term represents a randomly connected link between spots with similar tissue structures but not adjacent spatial locations or gene expression.

Accordingly, for the positive views, random graph augmentation methods proposed by You et al. [20] and the prior-based graph augmentation mentioned above are used. Specifically, the positive augmentation graphs ${\overset{ˆ}{G}}_{1},{\overset{ˆ}{G}}_{2}$ are derived from the original graph G through graph augmentation, where ${\overset{ˆ}{G}}_{1}=({\overset{ˆ}{X}}_{1},{\overset{ˆ}{A}}_{1})=\tau_{X}(X,A)$, and ${\overset{ˆ}{G}}_{2}=({\overset{ˆ}{X}}_{2},{\overset{ˆ}{A}}_{2})=\tau_{A}(X,A)$. For the negative view $\overset{˜}{G}$, attribute shuffling is used to generate it (Supplementary Section 1.1).

### 2.4 Domain feature extraction module

The domain feature extraction module comprises an encoder, a symmetrical decoder, and a subspace model. Accordingly, it is trained by minimizing local-global contrastive loss, local-subspace contrastive loss, and reconstruction loss (Fig. 1b).

#### 2.4.1 Graph representation learning

Graph contrastive learning [22], [23], [24] is a promising method in unsupervised representation learning, alleviating the heavy reliance on label information. The graph contrastive learning framework learns representations by maximizing the similarity between positive views while minimizing the similarity to negative views [16]. For instance, Deep Graph InfoMax (DGI) [22], a well-known model in this field, discriminates nodes with correct topology from those with corrupted topology [25]. This approach ensures that node representations capture as much global information as possible (Supplementary Section 1.1). GRAS4T utilizes DGI as its foundational framework, and further integrates task-specific information and an additional decoder structure.

Graph Convolutional Network (GCN) is used as the encoder of the GRAS4T. The single-layer encoder can be expressed as

(2) $$f(X,A)=\sigma \left(W_{e}X{\overset{ˆ}{D}}^{-\frac{1}{2}}\overset{ˆ}{A}{\overset{ˆ}{D}}^{-\frac{1}{2}}\right),$$

where $\overset{ˆ}{A}=A+I$ is the adjacency matrix with inserted self-loops **I** and $\overset{ˆ}{D}=\sum_{j}{\overset{ˆ}{A}}_{\cdot ,j}$ is the diagonal degree matrix. σ(⋅) is the nonlinear activation function (here we used Parametric Rectified Linear Unit [26]) and is applied column-wisely. $W_{e}\in R^{d^{\prime }\times d}$ ($d^{\prime }$ is the dimension of hidden feature) is a learnable weight matrix.

Contrary to DGI which learns spot embeddings by contrasting local and global information in the original view, task-aware embeddings are learned by contrasting the local and global information of augmentation views. Assume **H** is the representation of the input graph obtained from Eq. (2), and similarly, $\overset{˜}{H}$ is the representation of the negative view. The local-global contrastive loss can be formulated as

(3) $$L_{local-global}=-\frac{1}{2n}(\sum_{i=1}^{n}E_{\left(X,A\right)}\left[\log D\left({\overset{\to }{h}}_{i},{\overset{\to }{s}}_{1},{\overset{\to }{s}}_{2}\right)\right]+\sum_{j=1}^{n}E_{\left(\overset{˜}{X},\overset{˜}{A}\right)}\left[\log\left(1-D\left({\overset{\to }{\overset{˜}{h}}}_{j},{\overset{\to }{s}}_{1},{\overset{\to }{s}}_{2}\right)\right)\right]),$$

where ${\overset{\to }{h}}_{i}$ and ${\overset{\to }{\overset{˜}{h}}}_{j}$ are the *i*-th spot of **H** and *j*-th spot of $\overset{˜}{H}$, respectively. ${\overset{\to }{s}}_{1}$ and ${\overset{\to }{s}}_{2}$ denote the global representation of the augmented graphs ${\overset{ˆ}{H}}_{1}$ and ${\overset{ˆ}{H}}_{2}$ accordingly. That is, ${\overset{\to }{s}}_{1}=R({\overset{ˆ}{H}}_{1}),{\overset{\to }{s}}_{2}=R({\overset{ˆ}{H}}_{2})$, which represents the high-level summaries of augmented graph ${\overset{ˆ}{G}}_{1},{\overset{ˆ}{G}}_{2}$. Here, R(⋅) is a readout function and D(⋅,⋅,⋅) is a discriminator, which outputs probability scores (defined in Supplementary Section 1.1).

The structure of the encoder-decoder has the ability to preserve local spatial domain information [14], [11], [13]. In addition, the graph autoencoder provides local information while subspace-enhanced graph contrastive learning provides global and contextual information, which helps the GRAS4T model adapt to different downstream tasks. Here, we considered a GCN-based decoder symmetric with the encoder to reconstruct data **X**. The equation for the single-layer GCN-based decoder is

(4) $$g(H,A)=\sigma \left(W_{d}H{\overset{ˆ}{D}}^{-\frac{1}{2}}\overset{ˆ}{A}{\overset{ˆ}{D}}^{-\frac{1}{2}}\right),$$

where $W_{d}\in R^{d\times d^{\prime }}$ is also a learnable matrix. The latent representation **H** is fed into the decoder, which produces the reconstructed data $X^{\prime }$.

To obtain an effective latent representation, the reconstruction loss between **X** and $X^{\prime }$ is measured using mean-square error. The reconstruction loss is defined as

(5) $$L_{recon}=\frac{1}{n}{\left\|X-X^{\prime }\right\|}_{F}^{2},$$

where ${\left\|\cdot \right\|}_{F}$ denotes the Frobenius norm, i.e., ${\left\|X\right\|}_{F}={\left(\sum_{ij}|x_{ij}{|}^{2}\right)}^{1/2}$.

#### 2.4.2 Subspace analysis

Subspace analysis [27], [28], [29], [30] is an important unsupervised learning method that has achieved great success in bioinformatics, such as cell-type clustering in single-cell transcriptomics [31], [32] and spatial domain identification in spatial transcriptomics [33]. This method relies on the assumption that data distribution forms a union of subspace [27]. However, ST data is complex, and the relationships between spots are difficult to express linearly. To overcome these limitations, we derived the self-expression matrix **C** through the low-dimensional representation **H** of the input graph G. More specifically, the self-expression matrix **C** is obtained by solving the following optimization problem

(6) $$\min_{C}\frac{1}{2}{\left\|\Phi (H){\overset{ˆ}{D}}^{-\frac{1}{2}}\overset{ˆ}{A}{\overset{ˆ}{D}}^{-\frac{1}{2}}C-\Phi (H)\right\|}_{F}^{2}+\frac{\beta }{2}{\left\|C\right\|}_{F}^{2},$$

where $\Phi :R^{m}\to H$ is a mapping from the input space to the reproducing kernel Hilbert space H and *β* is the balance hyperparameter. This optimization problem has a closed-form solution, which is computationally efficient compared to iterative numerical methods such as the alternating direction method of multipliers [34]. Meanwhile, a post-processing strategy was designed to better identify closely connected co-domain nearest neighbors and remove residual noise from the ST data. To elaborate, we designed a filtering matrix **M** such that $M_{i,j}=1$ if $C_{i,j}$ is among the top *k* values in the *j*-th column of matrix **C** and $M_{i,j}=0$ otherwise. We then multiplied this filtering matrix with the self-expression matrix **C** to derive the subspace co-domain neighbor matrix $C^{⁎}$. More details of this subspace module can be found in the Supplementary Section 1.2.

Once the self-expression coefficient matrix **C** is obtained, a reconstructed representation containing local spatial information can be computed, i.e., $Z_{1}={\overset{ˆ}{H}}_{1}C,Z_{2}={\overset{ˆ}{H}}_{2}C$ (the process can be considered as linearization [35]). Given that spots within the same subspace tend to belong to the same domain, the reconstructed representation encapsulates local information specific to that domain. We compared the reconstructed representation $Z_{1}$ and $Z_{2}$, which include neighbor information, with the representation **H** and $\overset{˜}{H}$, respectively. These comparisons led to the formulation of the following local-subspace contrastive loss, defined as

(7) $$L_{subspace}=-\frac{1}{2n}(\sum_{i=1}^{n}E_{\left(X,A\right)}\left[\log D\left({\overset{\to }{h}}_{i},{\overset{\to }{z}}_{1i},{\overset{\to }{z}}_{2i}\right)\right]+\sum_{j=1}^{n}E_{\left(\overset{˜}{X},\overset{˜}{A}\right)}\left[\log\left(1-D\left({\overset{\to }{\overset{˜}{h}}}_{j},{\overset{\to }{z}}_{1j},{\overset{\to }{z}}_{2j}\right)\right)\right]),$$

where ${\overset{\to }{z}}_{1i}$ is the *i*-th spot of reconstructed representation $Z_{1}$, which denote the contextual information of spot *i* from augmented graph ${\overset{ˆ}{G}}_{1}$. Similarly, ${\overset{\to }{z}}_{2i}$ carries the same meaning for the augmented graph ${\overset{ˆ}{G}}_{2}$.

#### 2.4.3 Overall loss function

Combining Eq. (3), Eq. (5), and Eq. (7), GRAS4T optimized the following loss function

(8) $$L_{total}=L_{global}+\lambda_{1}\cdot L_{subspace}+\lambda_{2}\cdot L_{recon},$$

where $\lambda_{1}$ and $\lambda_{2}$ are trade-off hyperparameters that control the balance between the contributions of different modules.

### 2.5 Downstream tasks

*Domain identification.* GRAS4T adopted two classical clustering methods to cluster low-dimensional representation, namely the mclust algorithm from the mclust R package [5] and the Louvain algorithm from the scanpy package [36]. mclust and Louvain show advantages in datasets with different distributions. Both clustering methods include automated parameter selection functions and can be effectively applied to both annotated and unannotated datasets.

*Trajectory inference.* Based on the clustering results of GRAS4T, the trajectory inference analysis was performed using the learned low-dimensional representations. Particularly, the Partition-based Graph Abstraction (PAGA) [37] method from the scanpy package was applied for this analysis.

*Differential expression analysis.* To identify domain-specific marker genes from the differential gene expression analysis across different domains, the ‘*correlation_matrix*’ and ‘*rank_genes_groups_dotplot*’ methods in the scanpy package were used. When using the ‘*rank_genes_groups_dotplot*’ method, ‘min_logfoldchange’ is set to 2.

### 2.6 Hyperparameter setting and evaluation metrics

GRAS4T is implemented via PyTorch 1.8.0. In the experiments, the selection of nearest neighbor hyperparameters $k_{S}$ and $k_{E}$ depends on the number of spots or cells. In general, the larger the number of spots or cells, the larger the values for $k_{S}$ and $k_{E}$. Hyperparameters $k_{S}$ and $k_{E}$ are typically set to the same value. By default, we set $k_{S}=k_{E}=10$, but these values can be adjusted based on the number of spots or cells. For example, in the mouse olfactory bulb dataset, we set $k_{S}=k_{E}=50$. The balance hyperparameter *α*, which adjusts the weight between the two nearest neighbor graphs, is tuned according to the downstream clustering task. By default, this hyperparameter is set to 0.5, but for the cell type identification in the mouse hippocampus dataset, it is set to 0.7. The default graph augmentation methods for GRAS4T are ‘mask’ for nodes and ‘HS_image’ for edges. When the ST data does not contain an image, the classical edge augmentation method ‘edge’ can be used instead. This method randomly disconnects two connected nodes and randomly connects two unconnected nodes [38]. In our study, the encoder is configured as a single-layer GCN by default, with a low-dimensional embedding dimension of 64. The decoder is consistent with the encoder structure. Although GRAS4T allows adjustments to the number of network layers and the dimensions of hidden layers, it is recommended to limit the GCN layers to a maximum of 3. Going beyond this recommendation often results in an over-smoothing effect, leading to poor performance [39]. GRAS4T is optimized by the Adam optimizer [40] with a default learning rate of 0.001. It alternates the model training and subspace optimization every 100 iterations until the maximum number of iterations is reached, which is set to 500 by default. For the subspace module, the default hyperparameters provided by Cai et al. [29] were referenced. For example, the hyperparameter *β* is set to 1. The ratio of global loss, subspace loss, and reconstruction loss in the final loss function is 1:1:1 by default. In the Supplementary Section 2.3, we provide a detailed explanation of the key hyperparameters. All experiments were implemented using NVIDIA GeForce RTX 3070Ti GPU, Intel® Core (TM) i7-12700K CPU @ 3.60 GHz, and 64 GB memory.

We evaluated the performance of the competing methods by comparing the predicted or inferred labels with the gold-standard reference labels. Two widely used clustering evaluation metrics, the Adjusted Rand Index [41] (ARI) and the Normalized Mutual Information [42] (NMI), are employed. More information about the evaluation metrics can be found in Supplementary Section 2.4.

## 3 Results

### 3.1 Comparison GRAS4T with other methods on benchmark datasets

**Fig. 2:**
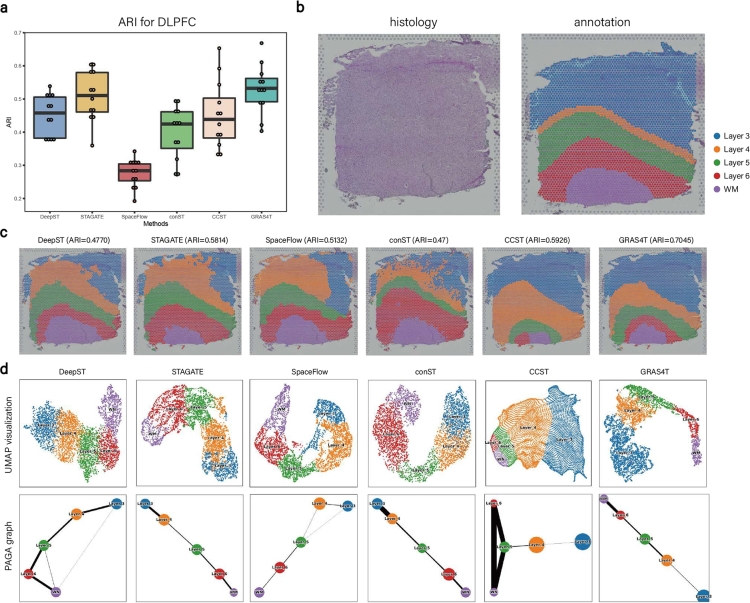
GRAS4T improved the accuracy of identifying layer structures within the DLPFC dataset compared to other methods. (a) Boxplot of ARI values across all sections of the DLPFC dataset for six methods. (b) The H&E image and manual annotation of slice 151672. (c) The spatial domains in six methods for slice 151672. (d) UMAP visualizations and PAGA graphs in six methods for slice 151672.

To demonstrate the effectiveness of GRAS4T, it was compared with other spatial domain identification methods including DeepST, STAGATE, SpaceFlow, conST, and CCST (Supplementary Table S2). By analyzing the DLPFC [43] dataset, GRAS4T demonstrated robust clustering capabilities and achieved the highest median ARI score across all 12 samples (Fig. 2a and Supplementary Figure S3), followed by STAGATE and DeepST, both based on graph autoencoder; SpaceFlow, conST, and CCST, utilizing DGI, showed smaller median ARI scores. Specifically, the median ARI score of GRAS4T surpassed that of STAGATE by 2.17% and outperformed CCST by 9.37%. As an example, in the DLPFC section 151672 (Fig. 2b), GRAS4T reported the best clustering accuracy (ARI=0.7045) while other models achieved ARI values ranged from 0.47 to 0.593 (Fig. 2c). It could identify domains consistent with the manually annotated structure. In contrast, CCST failed to separate the WM and Layer 6, while DeepST, STAGATE, SpaceFlow, and conST exhibited challenges in accurately identifying Layer 3 and Layer 4 (Fig. 2c). When evaluating the performance on the DLPFC dataset using NMI, we observed that GRAS4T's clustering results retained strong competitiveness (Supplementary Figures S3 and S7a). In addition, the removal of the subspace module led to a 2.36% decrease in the median ARI score of GRAS4T (Supplementary Figure S4a). This observation demonstrates the significant role of the subspace module in enhancing clustering performance by effectively capturing local information and positively influencing the overall representation learning model. Concurrently, adopting random edge perturbation for the edge augmentation portion in the positive view led to a 0.83% decline in the median ARI score of GRAS4T (Supplementary Figure S4b).

Notably, the visualization of the low-dimensional embedding of GRAS4T using Uniform Manifold Approximation and Projection (UMAP) [44] demonstrated spatial trajectories that were consistent with the developmental scenario. Using slice 151672 as an example, the UMAP of the embeddings successfully illustrated the developmental trajectory from WM to Layer 6. In contrast, CCST was unable to clearly depict the developmental hierarchy from WM to Layer 6. The well-revealed trajectory by GRAS4T's clustering results was further validated through the application of PAGA, which delineated the developmental trajectories across the various layers (Fig. 2d and Supplementary Figure S5). The results elucidated that among the algorithms evaluated, only the clustering results from GRAS4T, STAGATE, and conST led to PAGA graphs that unambiguously captured trajectories aligning with the anticipated developmental trajectories.

**Fig. 3:**
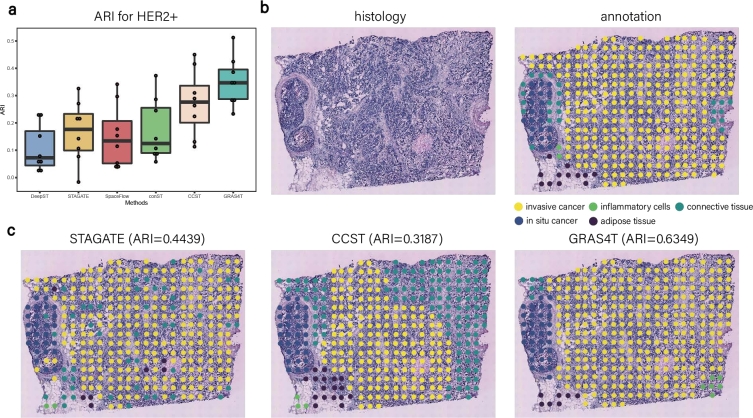
GRAS4T accurately identified spatial domains in the HER2+ dataset. (a) Boxplot of ARI values across all sections of the HER2+ dataset for all compared methods. (b) The H&E image and manual annotation for the A1 section. (c) Spatial domains of HER2+ dataset in (b) detected by STAGATE, CCST, and GRAS4T.

The efficacy of GRAS4T was further evaluated using the human HER2-positive breast tumor (HER2+) dataset [45], which used different spatial technologies (spatial transcriptomics) compared to the DLPFC dataset. We noticed a significant performance advantage of GRAS4T over all the other benchmark methods across eight slices (A-H) in the HER2+ dataset (Fig. 3a and Supplementary Figures S6). Specifically, the median ARI score of GRAS4T was 0.347, while that of all other methods fell below 0.3. Notably, GRAS4T's median ARI surpassed that of the second-ranked CCST method by 7.05%. STAGATE closely followed, while SpaceFlow and conST exhibited comparable performance. DeepST recorded the lowest median ARI score among the methods evaluated. For the A1 section, GRAS4T's identification aligned most closely with manual annotation shapes, achieving the highest ARI score (ARI=0.6349) as shown in Fig. 3b and c. Importantly, we accurately identified both invasive cancer and *in situ* cancer. In comparison, STAGATE mixed spots from different domains, whereas CCST, despite demonstrating more distinct cluster delineation, misidentified certain areas of invasive cancer as connective tissue. When employing NMI to assess the performance on the HER2+ dataset, GRAS4T demonstrated an advantage, indicating its efficacy in achieving robust spatial domain identification, even within the context of class imbalance in the ST dataset (Supplementary Figures S6 and S7b). We further tested the performance of six methods on a human breast cancer dataset from the 10X Visium platform. Compared with other spatial algorithms, GRAS4T showed a more comprehensive identification of the ductal carcinoma (DCIS) region, the lobular carcinoma (LCIS) region, and the invasive ductal carcinoma (IDC) region. Notably, GRAS4T achieved 0.83% higher median ARI than CCST, the second-ranked method (Supplementary Figure S8).

### 3.2 GRAS4T enhances domain separation with its block diagonal property

**Fig. 4:**
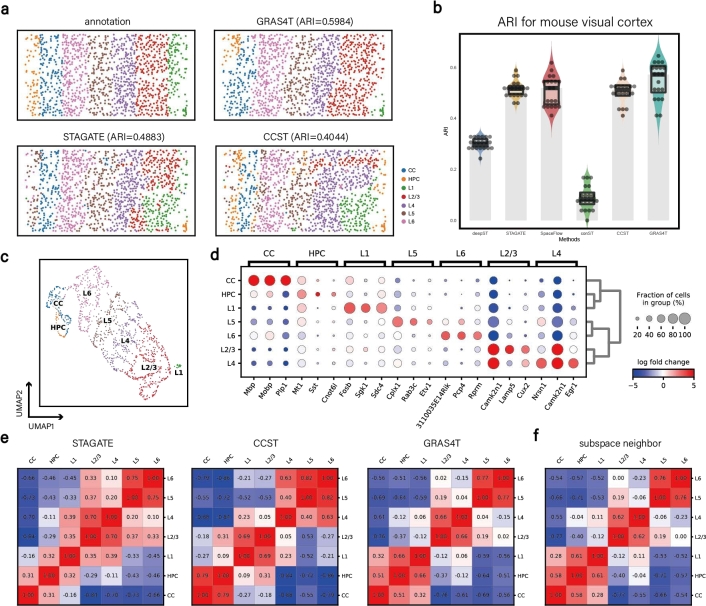
GRAS4T improved spatial domain identification results in the mouse visual cortex dataset and enhanced the separation between different spatial domains. (a) The manual annotation of mouse visual cortex and spatial domains detected by GRAS4T, STAGATE, and CCST. (b) Comparison of different methods by ARI pirate graph. (c) UMAP visualization using representations generated by GRAS4T. (d) Dotplot of the top 3 domain-specific genes for each domain. The size of the dots represents the proportion of cells expressing the gene. The color represents the average expression of the gene in the region where red reflects high expression and blue means low expression. (e) Heatmap of inter-domain Pearson correlation coefficients for STAGATE, CCST, and GRAS4T. (f) Heatmap of inter-domain Pearson correlation coefficients for subspace nearest neighbors in GRAS4T.

We used the mouse visual cortex dataset to further demonstrate GRAS4T's spatial domain separation capabilities. GRAS4T accurately identified the Hippocampus (HPC), Corpus Callosum (CC), and various layers of the cerebral cortex (L1, L2/3, L4, L5, and L6) in the mouse visual cortex. Compared to STAGATE and CCST, the domain regions identified by GRAS4T are more contiguous and highly consistent with manual labeling (Fig. 4a). The ARI pirate plots for the six methods demonstrated that GRAS4T had the highest median score. Meanwhile, STAGATE, CCST, and SpaceFlow had similar identification performance, while DeepST and conST had poor identification results (Fig. 4b). The UMAP visualization of the GRAS4T's embedding results demonstrated the trajectory from HPC, located in the deep part of the brain, to CC, and subsequently from L6 to L1, the most superficial layer of the cerebral cortex. This pattern of change accurately describes the differentiation process in the mouse brain (Fig. 4c). To gain insight into the gene expression profiles across distinct brain regions, we performed differentially expressed gene analysis for each domain's cells against all the other cells. The top three domain-specific genes per domain were illustrated in the dotplot (Fig. 4d). Our results revealed that the *3110035E14Rik* gene exhibits elevated expression in the L6 domain, while the *Cplx1* gene demonstrates high expression in the L5 domain. These results align with previous reports [46], bolstering the validity of our observations and providing additional support to the existing knowledge (Fig. 4d).

To unravel the inter-domain heterogeneity of gene expression, we calculated the inter-domain Pearson correlation coefficients. Compared to STAGATE, the coefficients-based heatmaps of GRAS4T and CCST showed greater dissimilarity in the non-diagonal sections. Taking GRAS4T, which has the highest ARI, as a benchmark, we found significant heterogeneity between CC, HPC, L1 and other domains (Fig. 4e). To examine the nearest neighbor coverage of individual spots, we assigned each spot to the most frequent class among its subspace nearest neighbors and generated a heatmap based on correlation coefficients. The results indicated that the correlation coefficient heatmap of subspace nearest neighbors exhibited a more block-diagonal pattern, suggesting that the spatial domains optimized by these neighbors likewise show stronger spatial heterogeneity (Fig. 4f). It is worth mentioning that our method consistently exhibited superior spatial heterogeneity in slice 151672 of DLPFC and A1 of HER2+, surpassing those of STAGATE and CCST (Supplementary Figure S9). In the following, we will show further advantages of GRAS4T in the task of deciphering spatial domains with more datasets. This performance was benchmarked against STAGATE and CCST, which are exemplary methods in the field of spatial domain identification.

### 3.3 GRAS4T captures co-domain neighbor to reveal finer-grained tissue structures

**Fig. 5:**
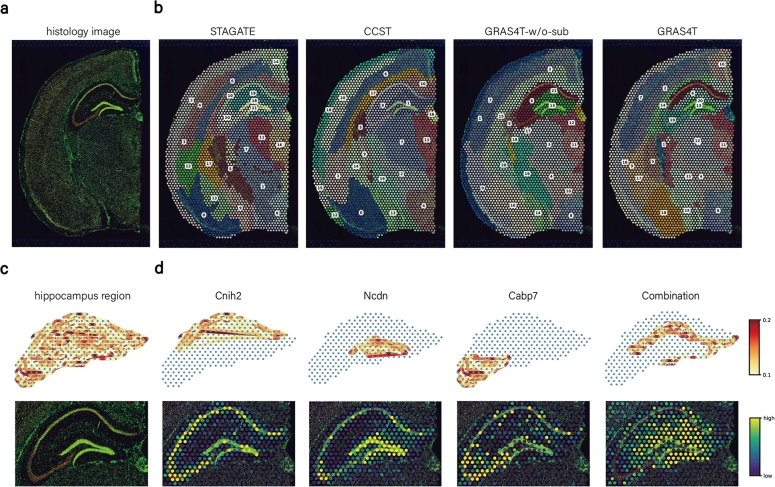
GRAS4T revealed refined spatial domains in the coronal section of an adult mouse brain. (a) The coronal mouse brain image stained with DAPI and Anti-NeuN. (b) Spatial domains generated by STAGATE, CCST, and GRAS4T without or with subspace module. (c) Visualization of the affinity matrix and H&E image for the hippocampal region. The nodes in the graph were reflected by the spots and the edges were determined by the values in the affinity matrix. (d) Visualizations of spatial domains identified by GRAS4T for the corresponding marker genes.

GRAS4T's capacity was assessed to select co-domain neighborhoods in complex biological tissues, particularly on the coronal mouse brain dataset (Fig. 5a). By utilizing the subspace module to capture localization information, we found that GRAS4T was able to identify smaller spatial domains in the hippocampus, such as CA1 (domain 6), CA3 (domain 19), and dentate gyrus (domain 13). By incorporating H&E images for graph augmentation, GRAS4T was able to identify regions that better matched the boundary information in morphological images. Although STAGATE could also describe small regions in the hippocampal part, it was not as effective in segmenting the boundaries of morphological images. Furthermore, CCST was unable to depict small spatial domains despite being able to identify smooth domain boundaries. It is worth noting that GRAS4T, when used without subspace selection of nearest neighbors, failed to identify domain 13 and domain 19, similar to the results obtained with CCST (Fig. 5b).

In order to delve deeper into the potential of GRAS4T's co-domain nearest neighbor selectivity to reveal finer tissue structures, we directed our investigation towards the hippocampal region and constructed a network heatmap utilizing spot location and subspace neighbor information (Fig. 5c-d). Examining the network of non-convex domain 6 and domain 20 revealed that GRAS4T selected spots that were not necessarily physically close. However, these spots were located within the same domain as nearest neighbor points, which facilitates the discovery of complex structural regions. The spatial domains identified by GRAS4T were clearly supported by known gene markers. For example, *Ncdn* was strongly expressed in dentate gyrus [47], while CA1 displayed pronounced expression of *Cabp7* [48]. Notably, we identified a unique gene combination (Combination = *Ddn*+*Camk2a*+*Slc1a2*-*Ncdn*-*Cnih2*) that accurately characterizes domain 6. Collectively, these findings demonstrated the remarkable ability of the GRAS4T subspace module in revealing finer-grained tissue structures. We further tested the performance of STAGATE, CCST, and GRAS4T on the mouse brain anterior&posterior dataset. Both GRAS4T and STAGATE showed great agreement with the reference (Supplementary Figure S10). In particular, GRAS4T can capture delicate structures like the dorsal and the ventral horn of the hippocampus region.

### 3.4 GRAS4T discerns the anatomical regions of tissue from ST data with different spatial resolutions

**Fig. 6:**
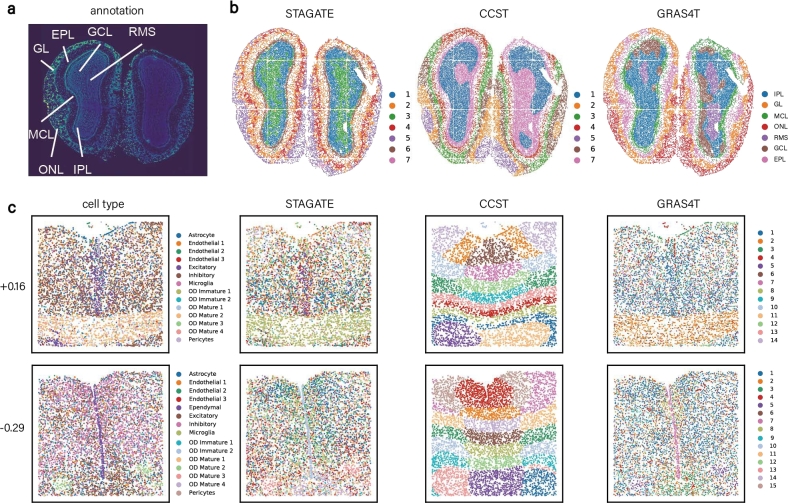
GRAS4T identifies spatial domains in ST datasets profiled by Stereo-seq and MERFISH platforms. (a) Laminar tissue of mouse olfactory bulb annotated in the DAPI-stained image. (b) Spatial domains detected by STAGATE, CCST, and GRAS4T. (c) Cell type and spatial domain identification by STAGATE, CCST, and GRAS4T in two slices of mouse hypothalamus dataset.

GRAS4T exhibited a remarkable performance in identifying complex spatial domains on datasets from both the 10X Visium and STARmap platforms. Furthermore, GRAS4T could also be adapted to other ST datasets with different spatial resolutions. A DAPI-stained image [49] was employed for the mouse olfactory bulb dataset generated by Stereo-seq [50] as a criterion for the spatial domain identification task (Fig. 6a). Although CCST effectively segmented the boundaries of each layer, it inappropriately divided the rostral migratory stream (RMS) into three domains and failed to delineate the inner layers, namely the mitral cell layer (MCL), internal plexiform layer (IPL), granule cell layer (GCL), and RMS. STAGATE was unable to clearly distinguish between the external plexiform layer (EPL) and glomerular layer (GL), resulting in mixed layer boundaries. In contrast, GRAS4T outperformed both STAGATE and CCST by more distinctly detecting the olfactory nerve layer (ONL), GL, EPL, and MCL, and successfully identifying the GCL (Fig. 6b). The performance of GRAS4T was further assessed in the mouse hypothalamus datasets generated by MERFISH [51]. Specifically, we performed a comparative analysis of STAGATE, CCST, and GRAS4T with reference cell types on 12 distinct slices of the dataset. The experimental results showed that GRAS4T remains competitive in identification of discontinuous domains (Supplementary Figure S11 and Table S4). For example, GRAS4T accurately identified oligodendrocytes (OD Mature2 domain) in the slice +0.16 and successfully captured the Ependymal domain in the slice -0.29 (Fig. 6c). Compared to STAGATE (ARI=0.0719 for +0.16, ARI=0.1167 for -0.29) and CCST (ARI=0.0494 for +0.16, ARI=0.0251 for -0.29), GRAS4T showed a high degree of consistency with the reference cell types (ARI=0.4641 for +0.16, ARI=0.4284 for -0.29). This superior performance in discontinuous domains is attributed to its ability to simultaneously consider spatial location and gene expression while constructing adjacency relationships.

## 4 Discussion and conclusion

The emergence of spatial transcriptomics has provided a spatial depiction of biological tissue structures and cellular functions. Naturally, the rich relationships among spots can be effectively captured through graph modeling. Learning graph representation is crucial for understanding the heterogeneity and similarities among spots. In this study, we developed a graph contrastive learning framework GRAS4T to perform spatial clustering or identify domains in spatial transcriptomics. GRAS4T simultaneously considers the local environment of spots, spatial domain features, and global information of the tissue section, thereby enabling precise delineation of spatial domains and adapting to various downstream tasks. Specifically, GRAS4T retains crucial information related to domain identification tasks in positive views through modality-aware graph augmentation. It also utilizes a subspace analysis module to adaptively capture the tissue microenvironment and spatial domain information. The modality-aware graph augmentation and subspace analysis strategy result in the effective separation of inter-domain regions and the precise capture of co-domain neighbors. We evaluated GRAS4T across a broad range of spatial transcriptomics datasets, encompassing different platforms and tissue types. The findings demonstrated the superiority of GRAS4T in identifying spatial domains.

One essential component of GRAS4T is the modality-aware graph augmentation in graph contrastive learning. Currently, the relationships among spots in the image (or morphology features) are employed to perform augmentation for the positive views where interactions and relationships between cells in spatially proximate neighborhoods are emphasized. For those ST datasets without image data associated, random graph augmentations are adopted to show their effectiveness in augmenting positive views. We noted that cells within the inferred subspace modules generally show similar gene expression profiles or furthermore potentially similar cell states or phenotypic characteristics. Therefore, they are well-suited for positive view augmentation. Furthermore, interactions between cells along spatial gradients or domain boundaries could serve as another angle for graph augmentation. Other studies also suggest seeking cells that shared similar biological characteristics or annotations (domains) or functional modules within the same datasets or across replicates [52], [53]. In addition, for spatial datasets with temporal series, creating positive views that capture similar patterns or dynamic changes over times could be an effective method for graph augmentation. These merit further investigations for both graph augmentation and the integration of multiple slices of data.

GRAS4T incorporates a subspace module with a graph contrastive learning framework, leveraging the strengths of both machine learning and deep learning approaches. Indeed, the combination of machine learning and deep learning is an interesting topic in the AI field [54], [55], [56]. A representation learning framework is required to produce a well-structured data representation that is suitable for subspace analysis (i.e., the representations in the latent feature space approximately lie in a union of subspaces) [57]. Concurrently, the representation with subspace structure can enhance the interpretability of deep learning models and mitigate the risk of model collapse [58]. Here, we use the self-expression matrix derived from the subspace to reconstruct the low-dimensional representations of the positive views through a one-step linearization [35] and to obtain the representations of the microenvironments that contain the same domain tissue. Meanwhile, we provide a post-processing tool that masks the self-expression matrix based on the magnitude of self-expression values [59]. This procedure effectively severs links between target points and non-domain counterparts, efficiently eliminating noise. Notably, this post-processing method can be characterized as an avenue for capturing the core cell set, facilitating the alignment and integration of multiple ST slices.

Although GRAS4T demonstrates its potential in spatial domain identification, it currently cannot directly assess the contribution of individual genes to specific spatial domains, relying instead on additional tools for post-hoc analysis. This reliance may complicate the analytical framework and limit the interpretability of the results. As such, this would be part of future work for improving the GRAS4T. Another challenge with GRAS4T is the cubic time complexity inherent in most subspace-based methods, which may hinder their effective application to large-scale datasets. As spatial transcriptomics datasets continue to grow, especially with high-resolution platforms, this computational complexity could become a major bottleneck. In future work, we plan to adopt an anchor sampling mechanism to overcome this limitation.

In conclusion, this study introduces GRAS4T, an innovative graph contrastive learning framework designed to effectively identify spatial domains in spatial transcriptomics. GRAS4T integrates local spot information, contextual domain information, and global tissue information, enhancing its effectiveness and versatility in downstream applications. Central to GRAS4T's efficacy is the modality-aware graph augmentation and the subspace module, both of which contribute to the effective separation of distinct spatial domains and the accurate identification of co-domain neighbors. Through a detailed discussion about GRAS4T and extensive experiments on different ST data, we demonstrate that GRAS4T is an ideal framework with extensibility.

## CRediT authorship contribution statement

**Yang Gui:** Writing – original draft, Visualization, Validation, Software, Methodology, Investigation, Formal analysis, Data curation. **Chao Li:** Writing – review & editing, Resources, Investigation, Conceptualization. **Yan Xu:** Writing – review & editing, Supervision, Resources, Project administration, Investigation, Conceptualization.

## Declaration of Competing Interest

The authors declare that they have no conflicts of interest related to this research.

## Data Availability

The ST datasets supporting the findings of this study are all publicly available. (1) The DLPFC dataset is available at http://research.libd.org/spatialLIBD/. (2) The HER2+ dataset generated by spatial transcriptomics platform is accessed at https://github.com/almaan/her2st. (3) The mouse visual cortex dataset generated by STARmap is available at https://www.dropbox.com/sh/f7ebheru1lbz91s/AADm6D54GSEFXB1feRy6OSASa/visual_1020/20180505_BY3_1kgenes?dl=0&subfolder_nav_tracking=1. (4) The adult mouse brain dataset is accessed at https://www.10xgenomics.com/resources/datasets. (5) The Stereo-seq mouse olfactory bulb dataset is available at https://github.com/JinmiaoChenLab/SEDR_analyses/. (6) The MERFISH dataset is accessed at https://datadryad.org/stash/dataset/doi:10.5061/dryad.8t8s248. (7) The human breast cancer dataset is available at https://www.10xgenomics.com/resources/datasets. (8) The anterior and posterior sections of the mouse brain are accessed at https://www.10xgenomics.com/resources/datasets and the Allen Brain Atlas reference is available at https://mouse.brain-map.org/static/atlas. All source codes used in our experiments have been deposited at https://github.com/Lab-Xu/GRAS4T.

## Appendix A Supplementary material

MMC This supplementary file contains a detailed description of the main techniques involved in GRAS4T, the experimental settings used in this study, and additional experimental results. MMC
