# Supplementary material for: Spatial domains identification in spatial transcriptomics using modality-aware and subspace-enhanced graph contrastive learning
Source: Comput Struct Biotechnol J. 2024 Oct 22;23:3703–13. doi: 10.1016/j.csbj.2024.10.029 (PMC11539238; doi:10.1016/j.csbj.2024.10.029)
Supplement: MMC — This supplementary file contains a detailed description of the main techniques involved in GRAS4T, the experimental settings used in this study, and additional experimental results. [file mmc1.pdf]

Supplementary Information

Spatial domains identification in spatial transcriptomics using modality-aware and subspace-enhanced graph contrastive learning

Yang Gui[a], Chao Li[b,*], and Yan Xu[a,*]

[a]School of Mathematics and Physics, University of Science and Technology Beijing, Beijing, 100083, China
[b]School of Statistics and Applied Mathematics, Anhui University of Finance and Economics, Bengbu, 233041, China
[*]Corresponding authors: xuyan@ustb.edu.cn (Yan Xu)

The supplementary material contains details of GRAS4T, experimental settings, and additional experimental results.

1 Detail of GRAS4T

In this section, we provided a detailed description of the two main techniques involved in the GRAS4T: graph contrastive learning and subspace analysis.

1.1 graph contrastive learning

Graph contrastive learning, an extension of self-supervised learning designed to learn robust representations from unlabeled graph data [1], consists of three main components:

- The graph data augmentation module is responsible for generating different views of a given graph. Graph data augmentation is essential in graph contrastive learning. When appropriate augmentations are applied, the corresponding prior is instilled and the model learns representations useful for downstream tasks by maximizing the consistency between the graph and its augmentations.
- The GNN-based encoder is used for computing representations. By training on data using Graph Neural Network (GNN), hidden representations are obtained, which contain rich graph structure information.
- The contrastive learning target is utilized for training the model. Contrastive learning obtains representations by maximizing mutual information between instances with similar semantic information, various pretext tasks can be constructed to enrich the supervision signals from such information [2].

The Deep Graph InfoMax (DGI) [3] is one of the most famous graph contrastive learning models. Given the undirected attribute graph $\mathcal{G} = \{\mathcal{V}, \mathcal{E}, \mathbf{X}\}$, where $\mathcal{V} = \{v_1, v_2, \cdots, v_n\}, (|\mathcal{V}| = n)$ is the set of nodes and $\mathcal{E}(|\mathcal{E}| = m)$ is the set of edges representing the relationship between node i and node j. The neighbors of the node v_i are denoted as $\mathcal{N}(v_i) = \{v_j \in \mathcal{V} \mid e_{i,j} \in \mathcal{E}\}$. $\mathbf{A} \in \mathbb{R}^{n\times n}$ is the adjacency matrix of $\mathcal{G}$ such that $\mathbf{A}_{i,j} = 1$ if $e_{i,j} \in \mathcal{E}$ and $\mathbf{A}_{i,j} = 0$ otherwise. $\mathbf{X} = [\boldsymbol{x}_1, \boldsymbol{x}_2, \cdots, \boldsymbol{x}_n] \in \mathbb{R}^{d\times n}$ is the feature matrix, where d is the dimension of the node.

The graph augmentation $\tau(\cdot)$ is applied in graph $\mathcal{G}$ to obtain the augmentation view. Many graph data augmentation methods have been developed, such as attribute (feature) masking, edge perturbation, and attribute shuffling [2, 3]. The DGI obtains a negative view through attribute shuffling, i.e. $(\tilde{\mathbf{X}}, \tilde{\mathbf{A}}) = \tau(\mathbf{X}, \mathbf{A})$. The attribute shuffling performs the column-wise shuffling on the feature matrix, we specify $\tau_{\mathbf{X}}^{neg}(\mathbf{X})$ for attribute shuffling as

$$\tau_{\mathbf{X}}^{neg}(\mathbf{X}) = \mathbf{X}\left[:, idx\right], \tag{1}$$

where idx is a randomly ordered list containing numbers from 1 to n. Attribute shuffling serves as an effective method for corrupting the graph structure by using column swapping.

DGI utilizes a one-layer Graph Convolutional Network (GCN) as the encoder of the model, the propagation of the GCN is depicted as

$$f(\mathbf{X}, \mathbf{A}) = \sigma\left(\mathbf{W}_e\mathbf{X}\hat{\mathbf{D}}^{-\frac{1}{2}}\hat{\mathbf{A}}\hat{\mathbf{D}}^{-\frac{1}{2}}\right), \tag{2}$$

where $\hat{\mathbf{A}} = \mathbf{A} + \mathbf{I}_N$ is the adjacency matrix with inserted self-loops and $\hat{\mathbf{D}} = \sum_j \hat{\mathbf{A}}_{\cdot,j}$ is the diagonal degree matrix. $\sigma(\cdot)$ denotes the nonlinear activation function such as the Parametric Rectified Linear Unit [4], applied column-wise. $\mathbf{W}_e \in \mathbb{R}^{d'\times d}$ (d' is the hidden feature number) is the learnable weight matrix.

The representation of input graph $\mathcal{G}$ and negative graph $\tilde{\mathcal{G}}$ are obtained by the GCN-based encoder, i.e. $\mathbf{H} = f(\mathbf{X}, \mathbf{A}), \tilde{\mathbf{H}} = f(\tilde{\mathbf{X}}, \tilde{\mathbf{A}})$.

DGI considers a noise-contrastive type objective with a standard binary cross-entropy (BCE) [5] loss between the samples from the positive view and the negative view. The loss function is defined as

$$\mathcal{L} = \frac{1}{n+m}\left(\sum_{i=1}^{n} \mathbb{E}_{(\mathbf{X},\mathbf{A})}\left[\log D\left(\vec{h}_i, \vec{s}\right)\right] + \sum_{j=1}^{m} \mathbb{E}_{(\tilde{\mathbf{X}},\tilde{\mathbf{A}})}\left[\log\left(1 - D\left(\vec{\tilde{h}}_j, \vec{s}\right)\right)\right]\right), \tag{3}$$

where $\vec{h}_i$ and $\vec{\tilde{h}}_j$ are the i-th spot of $\mathbf{H}$ and j-th spot of $\tilde{\mathbf{H}}$, respectively. $\vec{s}$ is high level representation, obtained by readout function $R(\cdot)$. $D(\cdot, \cdot)$ is defined as a discriminator, which outputs a probability score.

For the readout function $R(\cdot)$, DGI calculates a simple average of the representations for all nodes

$$R(\mathbf{H}) = \sigma\left(\frac{1}{n}\sum_{i=1}^{n} \vec{h}_i\right), \tag{4}$$

where $\sigma(\cdot)$ denotes the logisitic sigmoid nonlinearity. Here, the high-level representation obtained by the readout function is the high-level summaries of the graph, which contain global information.

For the discriminator $D(\cdot, \cdot)$, DGI scores the probability using a simple bilinear scoring function

$$D\left(\vec{h}_i, \vec{s}\right) = \sigma\left(\vec{h}_i^T \mathbf{\Theta} \vec{s}\right), \tag{5}$$

where $\mathbf{\Theta} \in \mathbb{R}^{d' \times d'}$ is a learnable scoring matrix and $\sigma(\cdot)$ is the logisitic sigmoid nonlinearity. Here, $D(\vec{h}_i, \vec{s})$ represents the consistency between representation of node v_i and graph level representation $\vec{s}$. Meanwhile, $1 - D(\vec{\tilde{h}}_j, \vec{s})$ represents the inconsistency between the representation of node $\tilde{v}_j$ for negative view and the graph level representation $\vec{s}$.

GRAS4T compares the two positive views with the original view and the negative view, respectively, and then the discriminator D needs to be redefined. Taking local-glocal contrastive loss as an example, GRAS4T computes the relationship between high-level representations $\vec{s}_1, \vec{s}_2$ and representation $\vec{h}_i$ by utilizing the discriminant $D(\cdot, \cdot, \cdot)$, which can be formulated as

$$D\left(\vec{h}_i, \vec{s}_1, \vec{s}_2\right) = \sigma\left(\vec{h}_i^T \mathbf{\Theta} \vec{s}_1 + \vec{h}_i^T \mathbf{\Theta} \vec{s}_2\right). \tag{6}$$

1.2 subspace analysis

Given a set of samples $\mathbf{X} = [\mathbf{X}_1, \cdots, \mathbf{X}_k] = [\boldsymbol{x}_1, \boldsymbol{x}_2, \cdots, \boldsymbol{x}_n] \in \mathbb{R}^{d \times n}$ drawn from an unknown union of k subspaces $\{S_i\}_{i=1}^k$ of unknown dimensions $d_i = dim(S_i), 0 < d_i < d, i = 1, \cdots, k$ and $\mathbf{X}_i$ is a set of samples from subspace S_i with sample size n_i, where $n = \sum_{i=1}^k n_i$ [6]. Based on the assumption of subspace analysis, each data point x_i is a linear combination of other data points in the same subspace, implying that the self-expressive matrix should be block diagonal in theory [7]. Further, the self-expressive model is a crucial component of subspace analysis [8]. More specifically, the key problem in subspace analysis involves obtaining the self-expressive matrix $\mathbf{C}$ by solving the following optimization problem

$$\min_C L(\mathbf{XC}, \mathbf{X}) + \beta \left\|\mathbf{C}\right\|_\xi, \ s.t. \ diag(\mathbf{C}) = 0, \tag{7}$$

where $\mathbf{X}$ is the dataset and β is the trade-off hyperparameter. The $diag(\mathbf{C}) = 0$ means that the diagonal of $\mathbf{C}$ is restricted to 0, which is used to prevent trivial solutions. The first term of the objective function reconstructs each sample from all other samples, indicating the difference between the input data and the represented data. The second term introduces a regularisation of the coefficient matrix. Based on a priori information, different norms ξ (including l_p-normalization, Frobenius-normalization, and nuclear norm regularizer) are used to obtain a self-expressive matrix with different reconstruction properties, such as sparsity, low-rank, and connectivity.

Due to the complexity of data in the real world, an increasing number of models are designed depending on the background of the problem and the assumptions about the data distribution [7, 9, 10]. Efficient kernel graph convolutional subspace clustering (EKGCSC) is a subspace clustering method introduced in [11]. EKGCSC considers the rich spatial structure information of the data and the non-linear relationships between points, the model seeks a block diagonal representation by minimization

$$\min_{\mathbf{C}} \frac{1}{2} \left\| \Phi(\mathbf{X}) \hat{\mathbf{D}}^{-\frac{1}{2}} \hat{\mathbf{A}} \hat{\mathbf{D}}^{-\frac{1}{2}} \mathbf{C} - \Phi(\mathbf{X}) \right\|_F^2 + \frac{\beta}{2} \left\| \mathbf{C} \right\|_F^2 . \tag{8}$$

Here, $\|\mathbf{C}\|_F$ denotes the Frobenius norm of matrix $\mathbf{C}$. $\Phi : \mathbb{R}^m \rightarrow \mathcal{H}$ is a mapping from the input space to the reproducing kernel Hilbert space $\mathcal{H}$. The elements in the kernel Gram matrix $\mathbf{K_{XX}}$ consist of the inner product of vectors in the Hilbert space $\mathcal{H}$, i.e. $[\mathbf{K_{XX}}]_{ij} = [\langle \Phi(\mathbf{X}), \Phi(\mathbf{X}) \rangle_{\mathcal{H}}] = \Phi(x_i)^T \Phi(x_j) = k(x_i, x_j)$. β is a balance hyperparameter. KGCSC set Gaussian kernel as kernel function k, i.e. $k(x_i, x_j) = \exp\left(-\gamma \left\| x_i - x_j \right\|^2\right)$. The optimization problem mentioned above has a closed-form solution that is computationally efficient compared to some iterative numerical methods such as the alternating direction method of multipliers [12]. The detail of the optimization process is provided in [11].

2 Experimental Settings

In this section, we described the datasets, baseline methods, and evaluation metrics in this paper.

2.1 datasets

We presented eight sets (38 sections in total) of challenging spatial transcriptomic (ST) datasets that were utilized in this paper. These datasets covered different platforms, tissues, sizes, and number of clusters, as detailed in Table S1. Four datasets were from the 10X Visium platform, encompassing various tissues and species: the human dorsolateral prefrontal cortex (DLPFC) [13], human breast cancer, mouse brain anterior&posterior, and coronal mouse brain. The DLPFC dataset was obtained by spatially analyzing gene expression in two pairs of 'spatial replicates' sections from three independent adult donors. Each pair consisted of two consecutive tissue sections 10 μm apart, with the second pair located 300 μm behind the first pair. In total, there were 12 slices, with each slice containing from 3,460 to 4,789 spots and capturing 33,538 genes. Furthermore, the coronal section of the adult mouse brain only had one slice which contained 2,903 spots with 32,285 genes. The one slice of the human breast cancer dataset contained 3,798 spots with 36,601 genes. Lastly, the mouse brain anterior&posterior dataset [14] contained 2,823 and 3,289 spots with 32,285 genes, respectively.

The remaining four datasets originated from various platforms and featured different spatial resolutions. Specifically, the human HER2-positive breast tumor (HER2+) dataset [15] was generated using the spatial transcriptomics platforms [16]. The HER2+ dataset comprised tumor tissue sections from eight HER2-positive patients (A-H). Researchers collected a total of 36 samples, extracting either three or six sections from each tumor for analysis. In this dataset, each slice contained from 167 to 659 spots and captured 14,861 to 15,661 genes. The mouse visual cortex dataset was generated based on STARmap [17], which contained 1,207 spots with 1,020 genes. The mouse olfactory bulb [18] with a spatial resolution of 14 μm, was produced by Stereo-seq [19], which contained 19,109 spots with 27,106 genes. Lastly, the mouse hypothalamus dataset was generated by MERFISH [20], a high-throughput and highly multiplexed method based on *in situ* hybridization technology. We selected all slices from one mouse, with each slice containing from 4,787 to 6,154 spots and capturing 155 genes.

Table S1: Detailed descriptions of the datasets used in this study.

Platform	Tissue	Section	Spot	Gene	Cluster	Reference
10X Visium	human dorsolateral prefrontal cortex	151507	4226	33538	7	[13]
		151508	4384	33538	7	
		151509	4789	33538	7	
		151510	4634	33538	7	
		151669	3661	33538	5	
		151670	3498	33538	5	
		151671	4110	33538	5	
		151672	4015	33538	5	
		151673	3639	33538	7	
		151674	3639	33538	7	
		151675	3673	33538	7	
		151676	3460	33538	7	
	human breast cancer	-	3798	36601	20	-
	mouse brain	coronal	2903	32285	-	-
		anterior	2823	32285	-	[14]
		posterior	3289	32285	-	
spatial transcriptomics	human breast cancer	A1	341	15045	5	[15]
		B1	269	15109	4	
		C1	167	15557	3	
		D1	255	15661	3	
		E1	534	15701	3	
		F1	659	14861	3	
		G2	402	15258	6	
		H1	530	15029	6	
MERFISH	mouse hippocampus	-0.29	5517	155	15	[20]
		-0.24	5543	155	15	
		-0.19	5803	155	15	
		-0.14	5926	155	15	
		-0.09	5557	155	15	
		-0.04	6154	155	14	
		+0.01	5338	155	15	
		+0.06	5343	155	15	
		+0.11	5070	155	15	
		+0.16	6067	155	14	
		+0.21	4787	155	15	
		+0.26	4832	155	15	
Stereo-seq	mouse olfactory bulb	-	19109	27106	7	[18]
STARMAP	mouse visual cortex	-	1207	1020	7	[17]

2.2 baseline methods

We compared GRAS4T with the following state-of-the-art spatial domain identification methods: DeepST, STAGATE, conST, SpaceFlow, and CCST. For algorithmic comparisons, we employed the default hyperparameters provided by these methods in their publications and codebases. To ensure fairness, we engaged hyperparameter tuning to rectify evidently problematic outcomes in the identification results. The software packages for all the baseline methods utilized in this paper are listed in Table S2.

Table S2: Detailed descriptions of the software packages of baseline methods.

Method	Language	Access	Reference
DeepST	python	https://github.com/JiangBioLab/DeepST	[21]
STAGATE	python	https:// github.com/zhanglabtools/STAGATE	[22]
conST	python	https://github.com/ys-zong/conST	[23]
SpaceFlow	python	https://github.com/hongleir/SpaceFlow	[24]
CCST	python	https://github.com/xiaoyeye/CCST	[25]

2.3 hyperparameter setting

We provide a more detailed explanation of the selection of graph construction hyperparameters k_S, k_E, and α.

The choice of k_S and k_E significantly impacts clustering results. A smaller k_S makes it easier to identify finer-grained clusters, but if k_S is too small, non-contiguous clusters may appear. A larger k_S leads to more contiguous clusters, but if k_S is too large, different clusters may not separate effectively. Similarly, a small k_E makes the model sensitive to local noise, while a large k_E can cause difficulties in separating clusters. Figure S1 displays the impact of different numbers of neighbors on spatial domain identification in the DLPFC and mouse olfactory bulb datasets. By default, we set the number of neighbors to $k_S = k_E = k$.

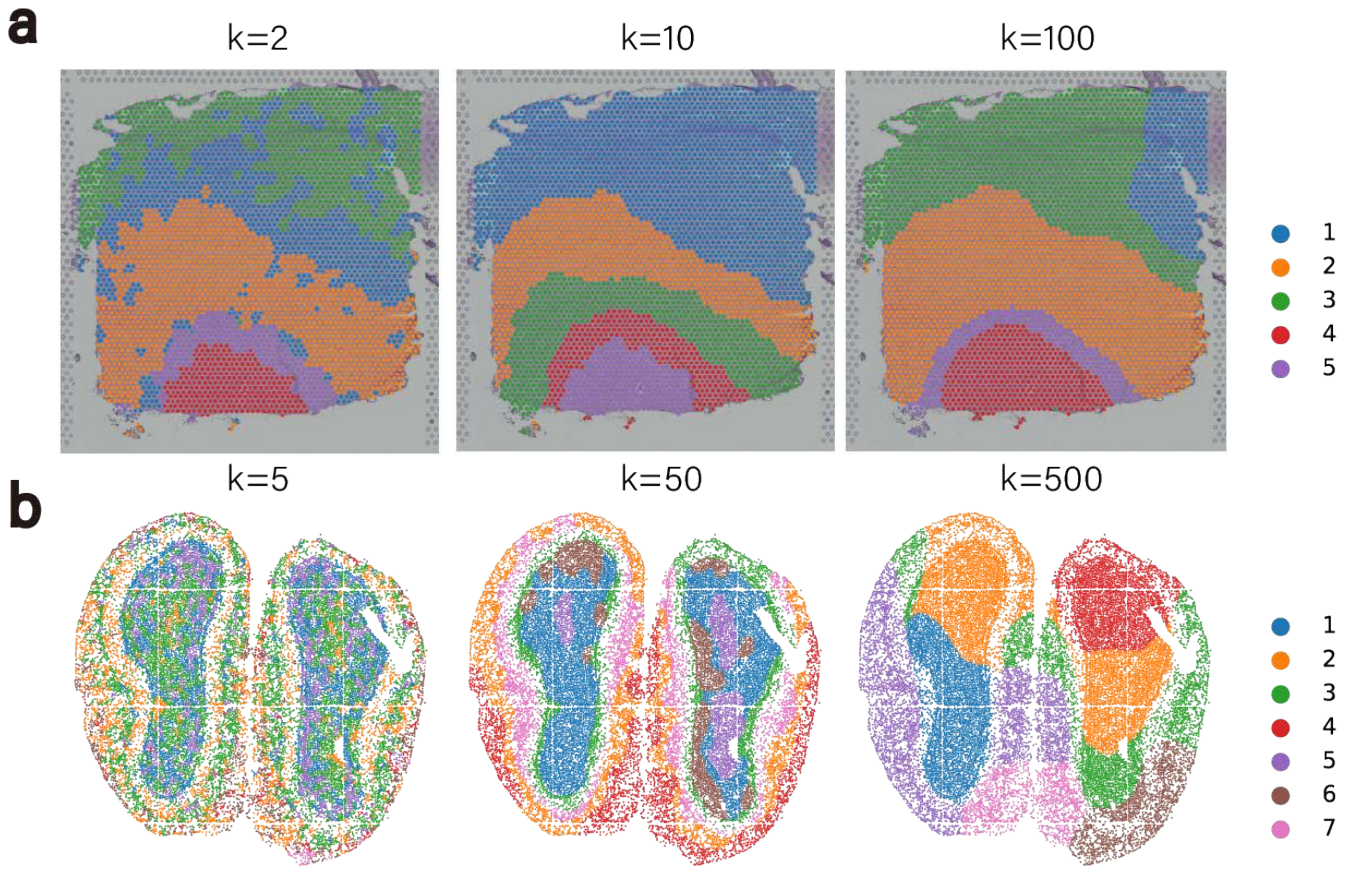

Figure S1: Spatial domain identification results with different numbers of neighbors. (a) Spatial domain identification results of GRAS4T for slice 151672 of DLPFC dataset with different numbers of neighbors set to 2, 10, and 100, respectively. (b) Spatial domain identification results of GRAS4T in the mouse olfactory bulb dataset with different numbers of neighbors set to 5, 50, and 500, respectively.

The choice of the hyperparameter α depends on the contiguity of the spots or cells in a region. If the spots or cells within a region are non-contiguous (e.g., the cell type identification task performed on the mouse hippocampus dataset), α should be closer to 1. Conversely, if the spots or cells within a region are contiguous (e.g., in the spatial domain identification task performed on the DLPFC dataset), α should be closer to 0. For the spatial domain identification task on slice 151672 of the DLPFC dataset, the ARI is relatively low when α is close to 1, but increases and remains at a high level as α approaches 0 (Supplementary Figure S2a). Whereas, for the cell type identification task on slice -0.29 of the mouse hippocampus dataset, the ARI is relatively low when α is close to 0, but increases significantly as α moves away from 0 (Supplementary Figure S2b).

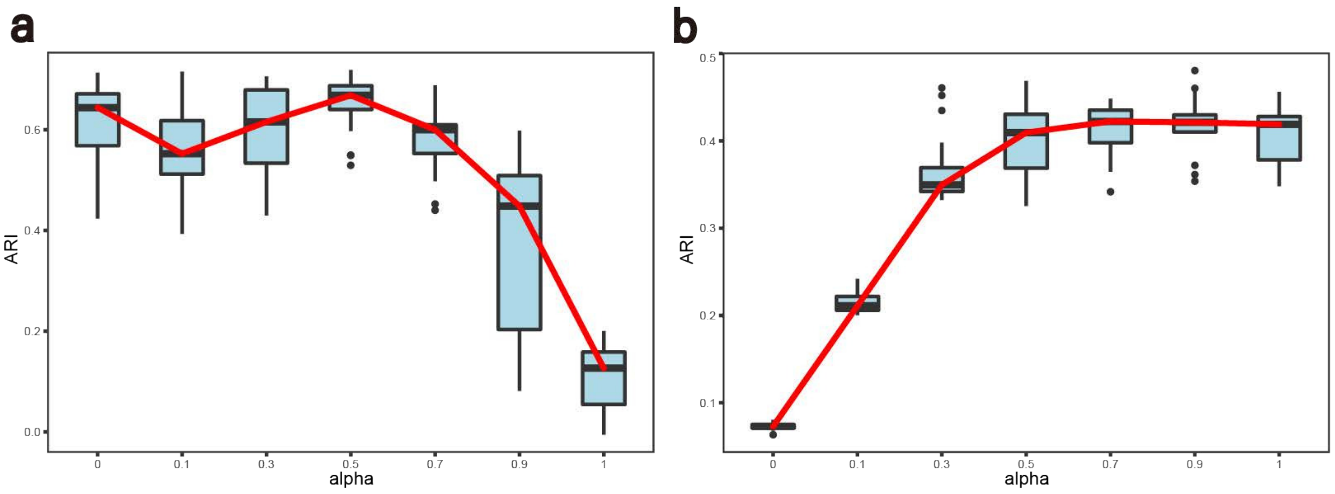

Figure S2: Robustness analysis of the hyperparameter α. (a) Results of spatial domain identification with different values of the hyperparameter α on slice 151672 of DLPFC dataset. (b) Results of cell type identification with different values of the hyperparameter α on slice -0.29 of the mouse hippocampus dataset.

2.4 evaluation metrics

We used two evaluation metrics, Adjusted Rand Index [26] (ARI) and Normalized Mutual Information [27] (NMI) to quantify the similarity between cluster labels and manual annotations. These two evaluation metrics measure the clustering performance by measuring the similarity between the clustering results $C^b = \{c_1^b, c_2^b, \cdots, c_k^b\}$ and the reference results $C^d = \{c_1^d, c_2^d, \cdots, c_k^d\}$. The cross-tabulation of C^b and C^d is shown in Table S3.

Table S3: The cross-tabulation of C^b and C^d.

	c_1	c_2	$\cdots$	c_k	sum
c_1	n_{11}	n_{12}	$\cdots$	n_{1k}	$n_{1\cdot}$
c_2	n_{21}	n_{22}	$\cdots$	n_{2k}	$n_{2\cdot}$
$\vdots$	$\vdots$	$\vdots$	$\ddots$	$\vdots$	$\vdots$
c_k	n_{k1}	n_{k2}	$\cdots$	n_{kk}	$n_{k\cdot}$
sum	$n_{\cdot 1}$	$n_{\cdot 2}$	$\cdots$	$n_{\cdot k}$	n

The ARI is a refined version of the Rand Index (RI). The RI treats the clustering results as a series of pairwise decisions and measures the clustering results based on the percentage of decisions that are correct. However, the RI cannot ensure that the score values for clustering results from randomized divisions are consistently near zero, this limitation led to the development of the ARI. The ARI score is calculated as

$$\text{ARI}\left(C^b, C^d\right) = \frac{r_0 - r_3}{\frac{1}{2}(r_1 + r_2) - r_3}, \tag{9}$$

where

$$r_0 = \sum_{i=1}^{k} \sum_{j=1}^{K} \binom{n_{ij}}{2}, \quad r_1 = \sum_{i=1}^{k} \binom{n_{i\cdot}}{2},$$

$$r_2 = \sum_{j=1}^{k} \binom{n_{\cdot j}}{2}, \quad r_3 = \frac{2 r_1 r_2}{n(n-1)}. \tag{10}$$

The NMI calculates the normalized similarity between two labels of the same data as follows

$$\mathrm{NMI}\left(C^b, C^d\right) = \frac{\sum_{i=1}^{k} \sum_{j=1}^{k} n_{ij} \log\left(\frac{n n_{ij}}{n_{i\cdot} \cdot n_{\cdot j}}\right)}{\sqrt{\left(\sum_{i=1}^{k} n_{i\cdot} \log\left(\frac{n_{i\cdot}}{n}\right)\right)\left(\sum_{j=1}^{k} n_{\cdot j} \log\left(\frac{n_{\cdot j}}{n}\right)\right)}}. \tag{11}$$

3 Additional Experimental Results

Here, we present additional experimental results. Figure S3 shows the ARI and NMI scores of the six spatial domain identification methods on the DLPFC dataset. Figure S4 displays the results of the ablation study on the DLPFC dataset. Figure S5 depicts the UMAP and PAGA visualization results of GRAS4T on the DLPFC dataset. Figure S6 shows the spatial domains of HER2+ detected by six methods. Figure S7 demonstrates the NMI scores of the spatial domain identification results on the DLPFC and HER2+ datasets. Figure S8 shows the spatial domain identification results of the six methods on the human breast cancer dataset. Figure S9 displays the heatmap of inter-domain Pearson correlation coefficients for the four methods on the slice 151672 of the DLPFC dataset and the slice A1 of the HER2+ dataset. Figure S10 shows the spatial domain identification results in the mouse brain anterior&posterior dataset detected by STAGATE, CCST, and GRAS4T. Figure S11 shows the spatial domain identification results on ST datasets with high spatial resolutions detected by STAGATE, CCST, and GRAS4T. Table S4 demonstrates the closeness of the three spatial domain identification methods to the reference cell types on the mouse hypothalamus datasets.

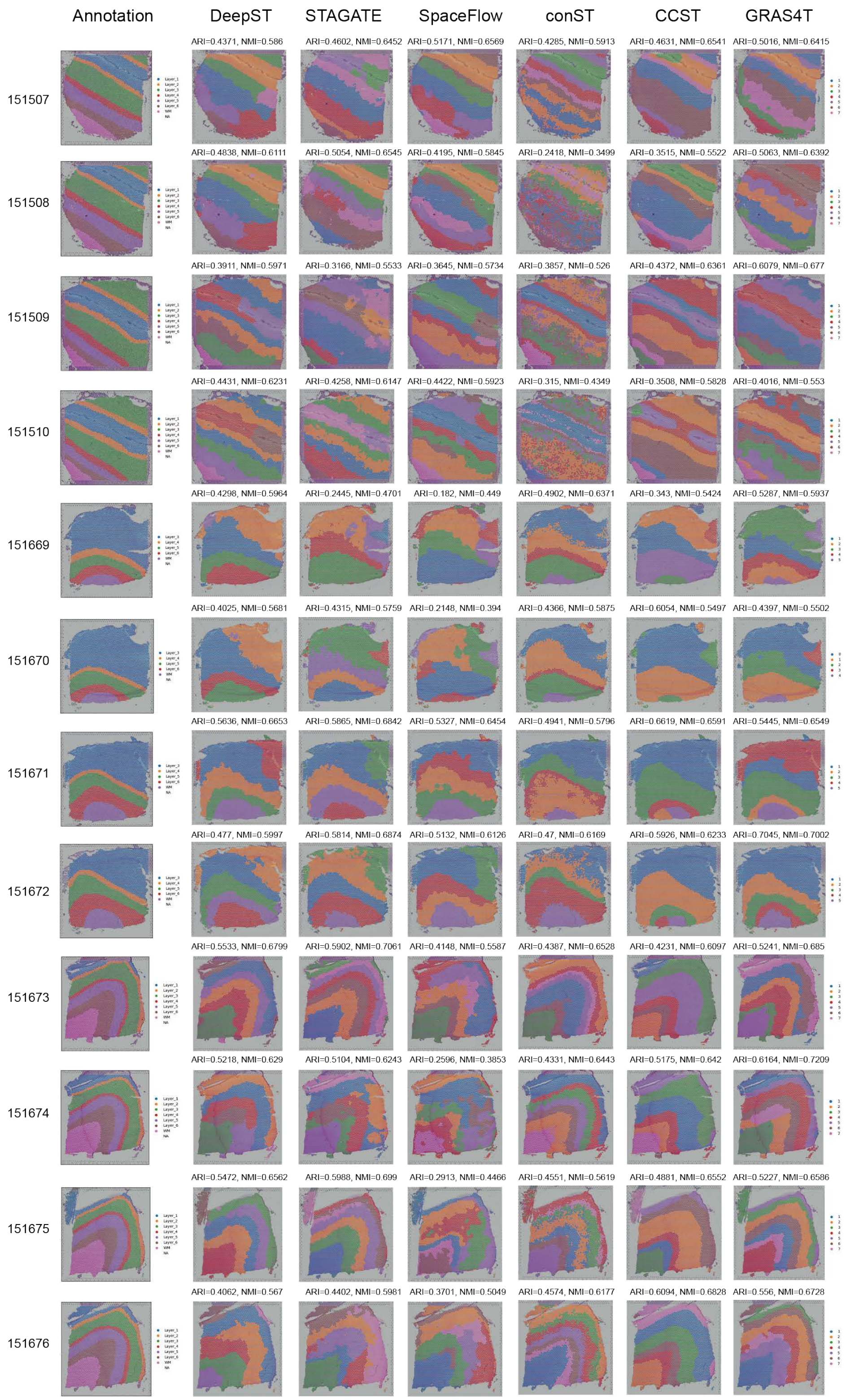

Figure S3: Manual annotations and comparison of spatial domains identified by DeepST, STAGATE, SpaceFlow, conST, CCST, and GRAS4T on the 12 slices of DLPFC dataset.

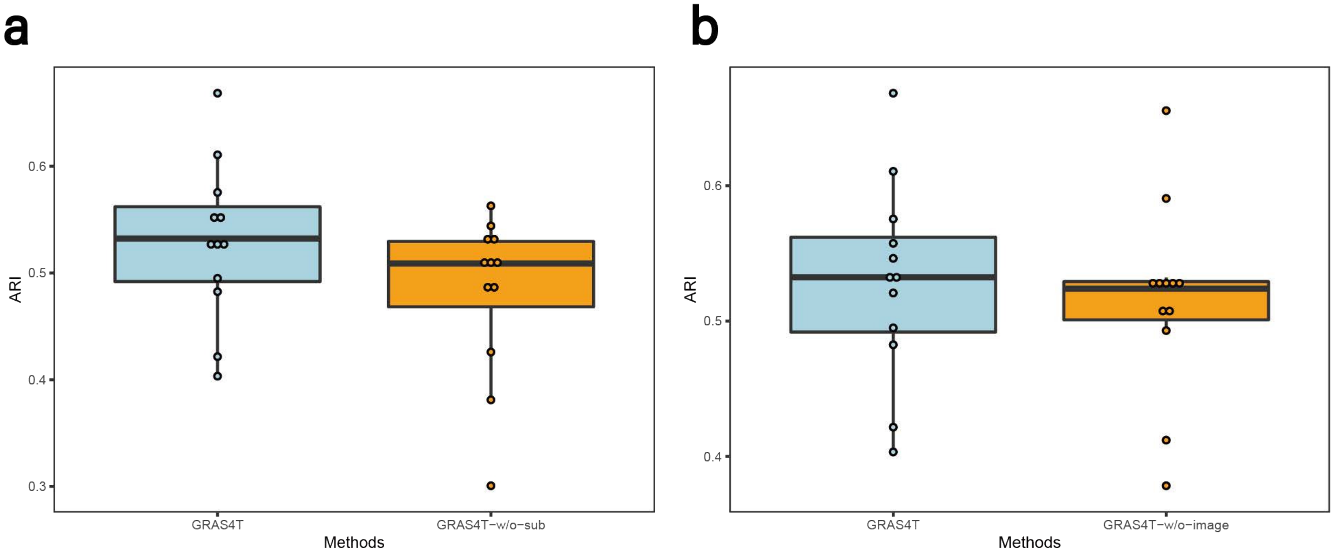

Figure S4: Ablation study of different components measured by ARI. (a) Comparison of ARI scores on DLPFC between GRAS4T and GRAS4T without subspace module. (b) Comparison of ARI scores on DLPFC between GRAS4T and GRAS4T without H&E image-based augmentation.

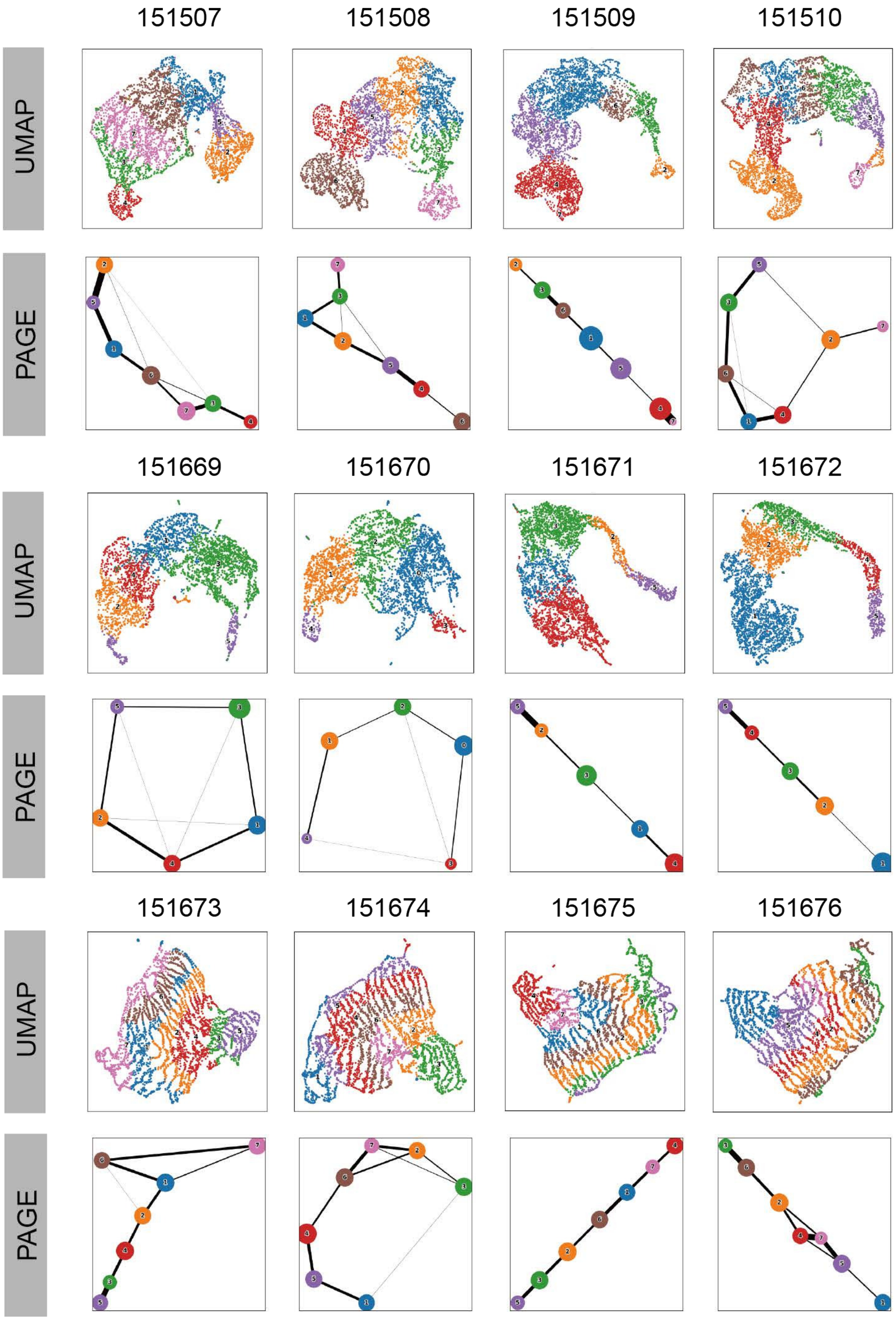

Figure S5: UMAP visualizations and PAGA graphs generated by GRAS4T on the 12 slices of DLPFC dataset.

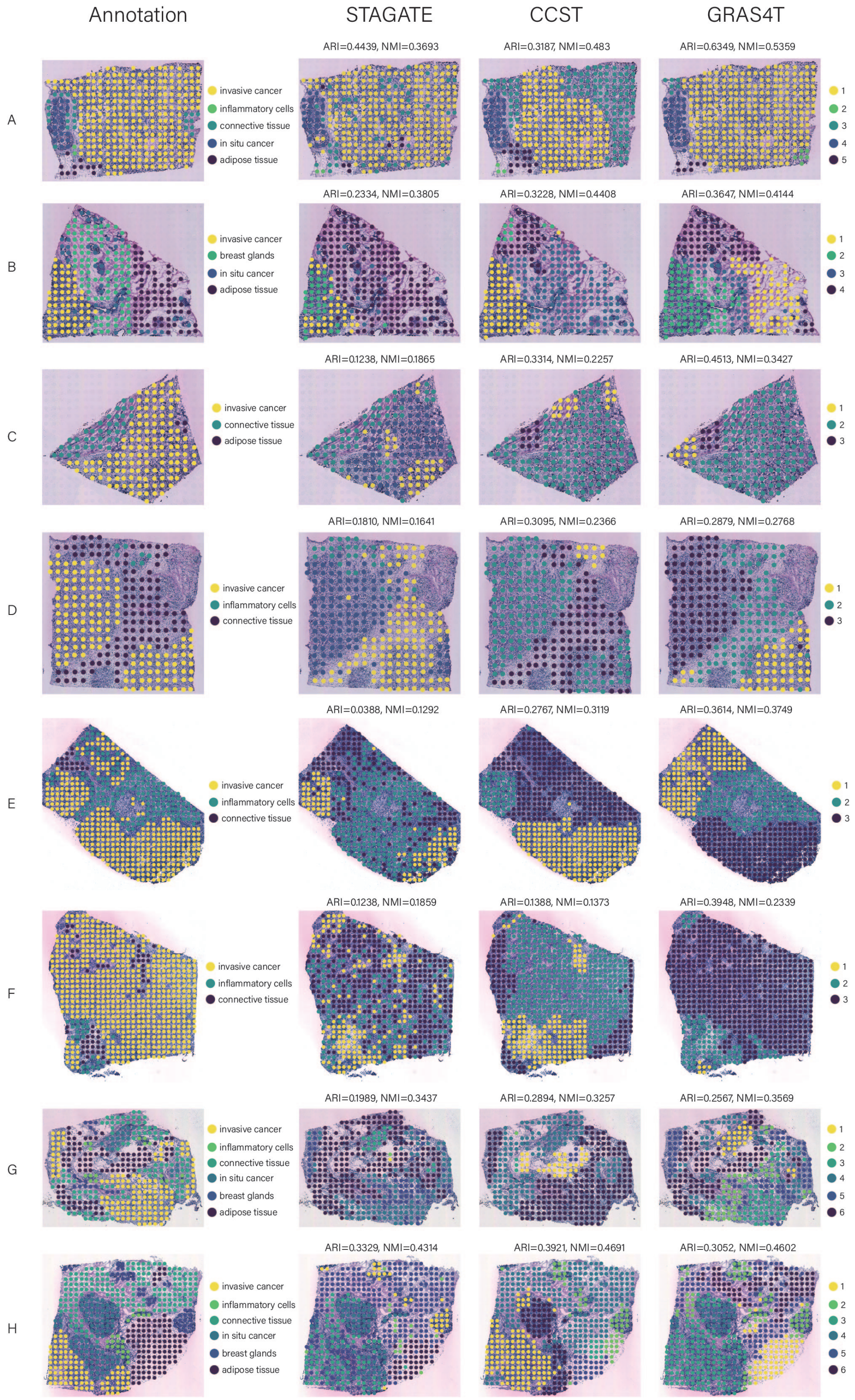

Figure S6: Manual annotations and comparison of spatial domains identified by STAGATE, CCST, and GRAS4T on the 8 slices of HER2+ dataset.

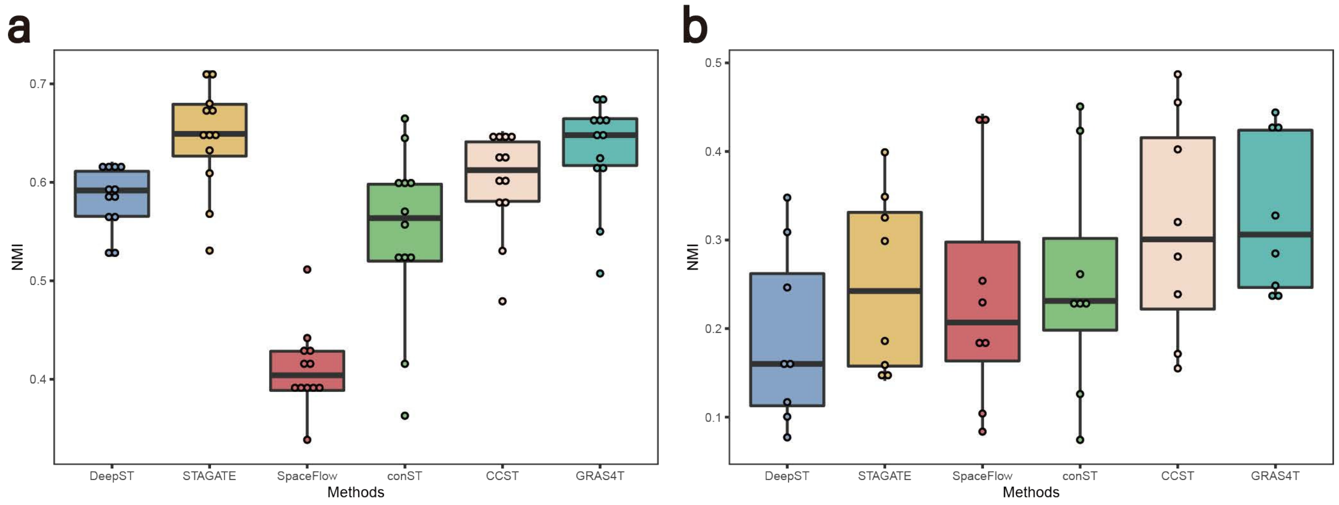

Figure S7: Spatial domain identification performance of six methods in terms of NMI. (a) Boxplot of NMI values for all methods across all slices of the DLPFC dataset. (b) Boxplot of NMI values for all methods across all slices of the HER2+ dataset.

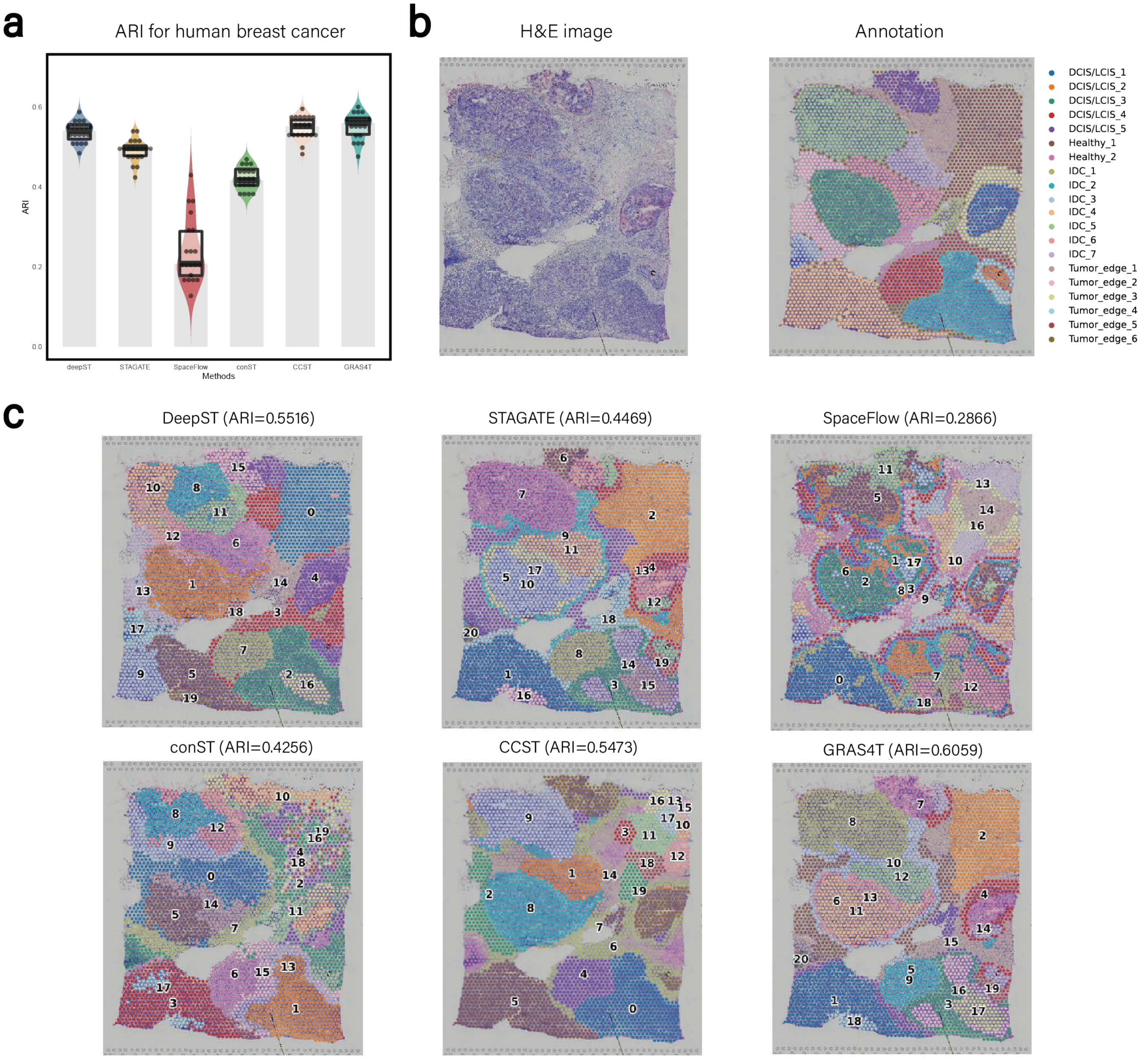

Figure S8: Comparison of spatial domains identified by DeepST, STAGATE, SpaceFlow, conST, CCST, and GRAS4T in the human breast cancer dataset. (a) Comparison of different methods by ARI pirate plot. (b) H&E image and manual annotation. (c)Visualizations of spatial domains in (b) identified by six methods.

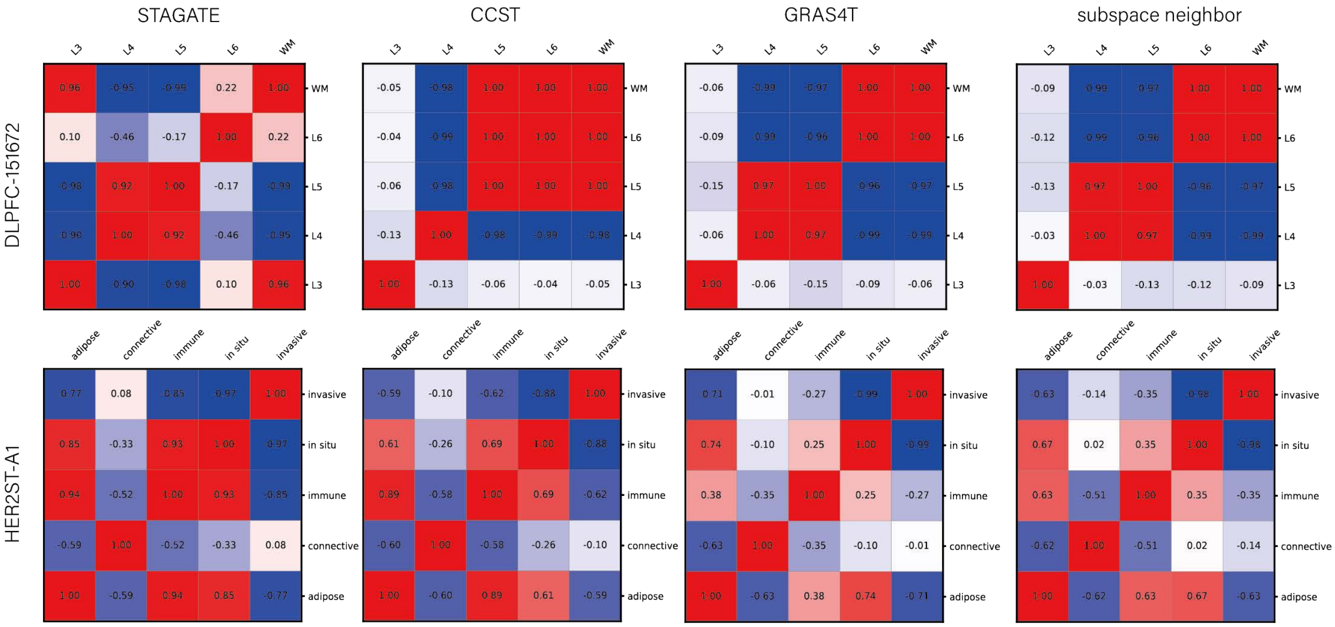

Figure S9: Heatmaps of inter-domain Pearson correlation coefficients for STAGATE, CCST, GRAS4T, and GRAS4T using subspace nearest neighbor adjustment.

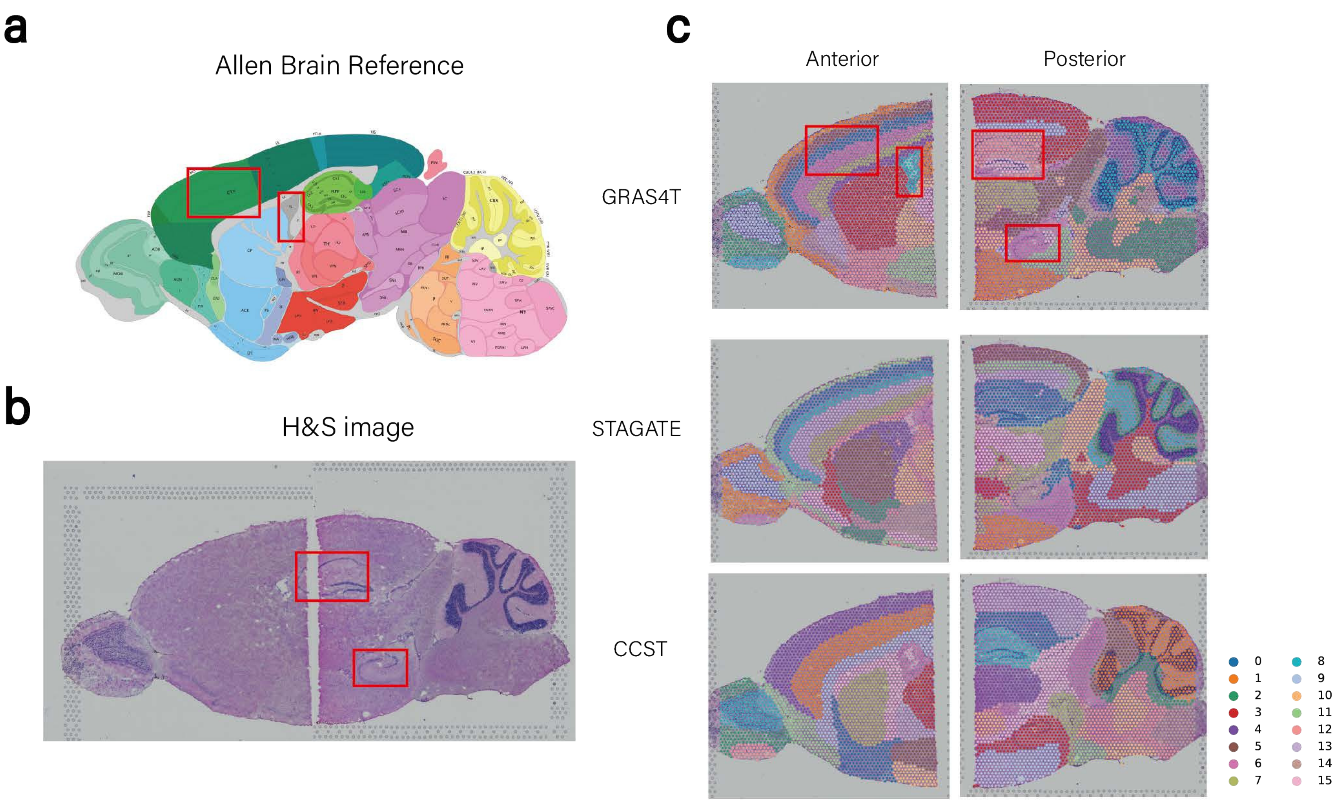

Figure S10: Spatial domain identification in the mouse brain anterior&posterior dataset. (a) Annotated brain section image from Allen Mouse Brain Atlas for reference. (b) H&E image of mouse brain anterior and posterior. (c) Spatial domains in (b) detected by GRAS4T, STAGATE, and CCST.

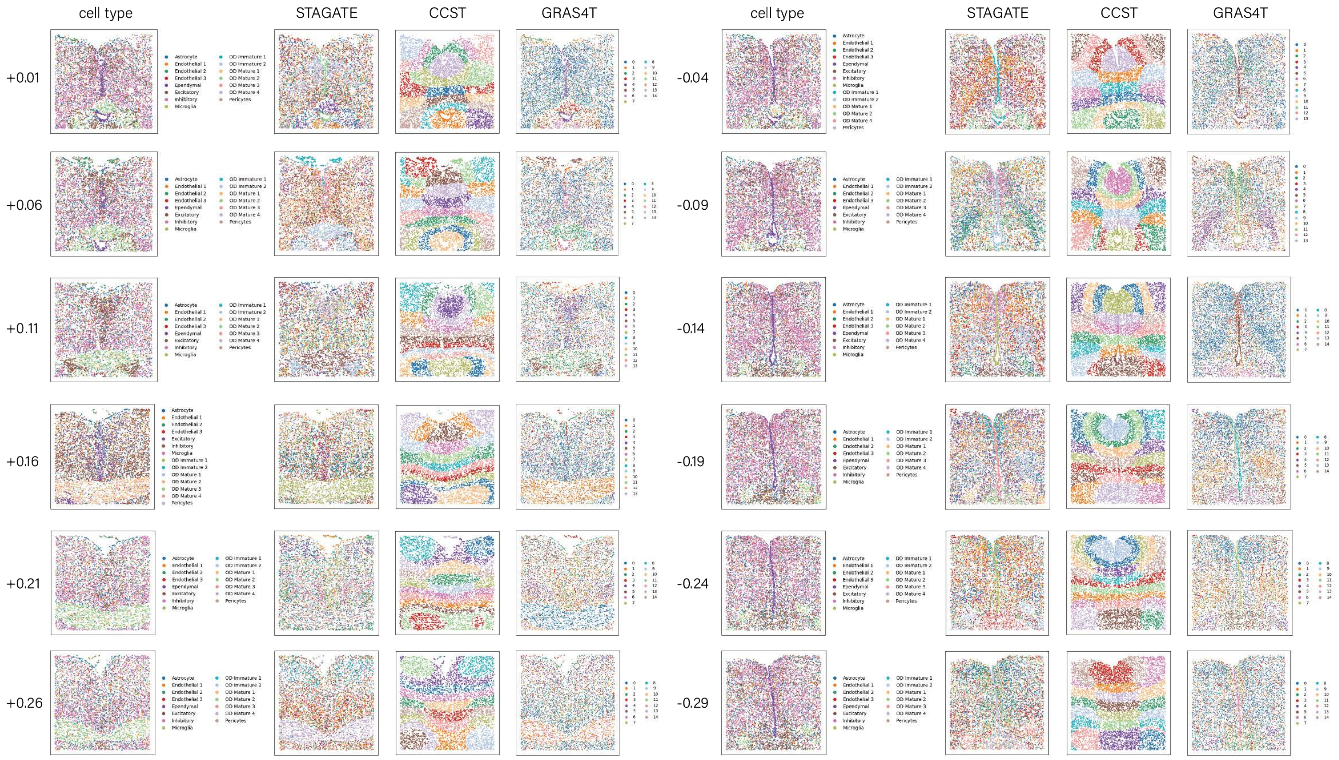

Figure S11: Cell types identified by STAGATE, CCST, and GRAS4T on the 12 slices of mouse hypothalamus datasets.

Table S4: The closeness (using ARI metrics) of the three spatial domain identification methods to the reference cell types on the mouse hypothalamus datasets.

slice	STAGATE	CCST	GRAS4T
+0.01	0.0826	0.0317	0.5005
+0.06	0.0682	0.0455	0.4418
+0.11	0.1078	0.0526	0.4495
+0.16	0.0719	0.0494	0.4641
+0.21	0.0264	0.0443	0.4206
+0.26	0.0451	0.0186	0.3780
-0.04	0.0805	0.0175	0.3818
-0.09	0.0403	0.0249	0.2784
-0.14	0.0754	0.0257	0.4711
-0.19	0.0708	0.0236	0.3970
-0.24	0.1530	0.0333	0.3863
-0.29	0.1167	0.0251	0.4284

References

1. Yuning You, Tianlong Chen, Yang Shen, and Zhangyang Wang. Graph contrastive learning automated. In *International Conference on Machine Learning*, pages 12121–12132. PMLR, 2021.

2. Yixin Liu, Ming Jin, Shirui Pan, Chuan Zhou, Yu Zheng, Feng Xia, and Philip Yu. Graph self-supervised learning: A survey. *IEEE Transactions on Knowledge and Data Engineering*, 2022.

3. Petar Velickovic, William Fedus, William L Hamilton, Pietro Liò, Yoshua Bengio, and R Devon Hjelm. Deep graph infomax. *ICLR (Poster)*, 2(3):4, 2019.

4. Kaiming He, Xiangyu Zhang, Shaoqing Ren, and Jian Sun. Delving deep into rectifiers: Surpassing human-level performance on imagenet classification. In *Proceedings of the IEEE International Conference on Computer Vision (ICCV)*, December 2015.

5. Yuning You, Tianlong Chen, Yongduo Sui, Ting Chen, Zhangyang Wang, and Yang Shen. Graph contrastive learning with augmentations. *Advances in neural information processing systems*, 33:5812–5823, 2020.

6. René Vidal. Subspace clustering. *IEEE Signal Processing Magazine*, 28(2):52–68, 2011.

7. Canyi Lu, Jiashi Feng, Zhouchen Lin, Tao Mei, and Shuicheng Yan. Subspace clustering by block diagonal representation. *IEEE transactions on pattern analysis and machine intelligence*, 41(2):487–501, 2018.

8. Shangzhi Zhang, Chong You, René Vidal, and Chun-Guang Li. Learning a self-expressive network for subspace clustering. In *Proceedings of the IEEE/CVF Conference on Computer Vision and Pattern Recognition*, pages 12393–12403, 2021.

9. Ehsan Elhamifar and René Vidal. Sparse subspace clustering: Algorithm, theory, and applications. *IEEE transactions on pattern analysis and machine intelligence*, 35(11):2765–2781, 2013.

10. Jufeng Yang, Jie Liang, Kai Wang, Paul L Rosin, and Ming-Hsuan Yang. Subspace clustering via good neighbors. *IEEE transactions on pattern analysis and machine intelligence*, 42(6):1537–1544, 2019.

11. Yaoming Cai, Zijia Zhang, Zhihua Cai, Xiaobo Liu, Xinwei Jiang, and Qin Yan. Graph convolutional subspace clustering: A robust subspace clustering framework for hyperspectral image. *IEEE Transactions on Geoscience and Remote Sensing*, 59(5):4191–4202, 2020.

12. Stephen Boyd, Neal Parikh, Eric Chu, Borja Peleato, Jonathan Eckstein, et al. Distributed optimization and statistical learning via the alternating direction method of multipliers. *Foundations and Trends (R) in Machine learning*, 3(1):1–122, 2011.

13. Kristen R Maynard, Leonardo Collado-Torres, Lukas M Weber, Cedric Uytingco, Brianna K Barry, Stephen R Williams, Joseph L Catallini, Matthew N Tran, Zachary Besich, Madhavi Tippani, et al. Transcriptome-scale spatial gene expression in the human dorsolateral prefrontal cortex. *Nature neuroscience*, 24(3):425–436, 2021.

14. Allen Institute for Brain Science. Allen brain atlas: Mouse brain. *https://mouse.brain-map.org/static/atlas*, 2008.

15. Alma Andersson, Ludvig Larsson, Linnea Stenbeck, Fredrik Salmén, Anna Ehinger, Sunny Z Wu, Ghamdan Al-Eryani, Daniel Roden, Alex Swarbrick, Åke Borg, et al. Spatial deconvolution of her2-positive breast cancer delineates tumor-associated cell type interactions. *Nature communications*, 12(1):6012, 2021.

16. Patrik L Ståhl, Fredrik Salmén, Sanja Vickovic, Anna Lundmark, José Fernández Navarro, Jens Magnusson, Stefania Giacomello, Michaela Asp, Jakub O Westholm, Mikael Huss, et al. Visualization and analysis of gene expression in tissue sections by spatial transcriptomics. *Science*, 353(6294):78–82, 2016.

17. Xiao Wang, William E Allen, Matthew A Wright, Emily L Sylwestrak, Nikolay Samusik, Sam Vesuna, Kathryn Evans, Cindy Liu, Charu Ramakrishnan, Jia Liu, et al. Three-dimensional intact-tissue sequencing of single-cell transcriptional states. *Science*, 361(6400):eaat5691, 2018.

18. Ao Chen, Sha Liao, Mengnan Cheng, Kailong Ma, Liang Wu, Yiwei Lai, Jin Yang, Wenjiao Li, Jiangshan Xu, Shijie Hao, et al. Large field of view-spatially resolved transcriptomics at nanoscale resolution. *BioRxiv*, 2021, 2021.

19. Ao Chen, Sha Liao, Mengnan Cheng, Kailong Ma, Liang Wu, Yiwei Lai, Xiaojie Qiu, Jin Yang, Jiangshan Xu, Shijie Hao, et al. Spatiotemporal transcriptomic atlas of mouse organogenesis using dna nanoball-patterned arrays. *Cell*, 185(10):1777–1792, 2022.

20. Jeffrey R Moffitt, Dhananjay Bambah-Mukku, Stephen W Eichhorn, Eric Vaughn, Karthik Shekhar, Julio D Perez, Nimrod D Rubinstein, Junjie Hao, Aviv Regev, Catherine Dulac, et al. Molecular, spatial, and functional single-cell profiling of the hypothalamic preoptic region. *Science*, 362(6416):eaau5324, 2018.

21. Jian Hu, Xiangjie Li, Kyle Coleman, Amelia Schroeder, Nan Ma, David J Irwin, Edward B Lee, Russell T Shinohara, and Mingyao Li. Spagcn: Integrating gene expression, spatial location and histology to identify spatial domains and spatially variable genes by graph convolutional network. *Nature methods*, 18(11):1342–1351, 2021.

22. Kangning Dong and Shihua Zhang. Deciphering spatial domains from spatially resolved transcriptomics with an adaptive graph attention auto-encoder. *Nature communications*, 13(1):1739, 2022.

23. Yongshuo Zong, Tingyang Yu, Xuesong Wang, Yixuan Wang, Zhihang Hu, and Yu Li. const: an interpretable multi-modal contrastive learning framework for spatial transcriptomics. *bioRxiv*, pages 2022–01, 2022.

24. Honglei Ren, Benjamin L Walker, Zixuan Cang, and Qing Nie. Identifying multicellular spatiotemporal organization of cells with spaceflow. *Nature communications*, 13(1):4076, 2022.

25. Jiachen Li, Siheng Chen, Xiaoyong Pan, Ye Yuan, and Hong-Bin Shen. Cell clustering for spatial transcriptomics data with graph neural networks. *Nature Computational Science*, 2(6):399–408, 2022.

26. William M Rand. Objective criteria for the evaluation of clustering methods. *Journal of the American Statistical association*, 66(336):846–850, 1971.

27. Alexander Strehl and Joydeep Ghosh. Cluster ensembles—a knowledge reuse framework for combining multiple partitions. *Journal of machine learning research*, 3(Dec):583–617, 2002.
